# Enhancing large language model clinical support information with machine learning risk and explainability: a feasibility study

**DOI:** 10.1186/s40635-026-00900-w

**Published:** 2026-04-21

**Authors:** Yu-Chang Yeh, Hsin-Yu Yang, Ching-Tang Chiu, Anne Chao, Yu-Chen Chuang, Wing-Sum Chan

**Affiliations:** 1 Departments of Anesthesiology, Information Technology Office, National Taiwan University Hospital, No 7, Chung Shan South Road, Taipei, Taiwan; 2 Information Technology Office, National Taiwan University Hospital, No 7, Chung Shan South Road, Taipei, Taiwan; 3 Department of Anesthesiology, Far Eastern Memorial Hospital, Nanya S. Rd, No. 21, Sec. 2, New Taipei City, Taiwan

**Keywords:** Critical care, Machine learning, Large language model, Generative artificial intelligence, Clinical support system

## Abstract

**Background:**

**C**urrent machine learning (ML) prediction models offer limited guidance for individualized actionable management. Large language models (LLMs) can transform ML model-predicted risk estimates with Shapley Additive Explanations (SHAP) into clinically meaningful support information, yet the added value of incorporating ML-derived data and the relative performance of different LLMs remain uncertain. To address these gaps, we used our previously developed IMPACT framework to evaluate the quality of LLM-generated outputs.

**Methods:**

In this retrospective analysis of MIMIC-IV v3.1 intensive care unit (ICU) admissions, we applied a previously developed XGBoost model to estimate ICU mortality risk and derive corresponding SHAP values. GPT-4o transformed the predicted mortality risk, clinical predictors, and their SHAP values into risk interpretation, recommended examinations and management. The primary analysis examined whether augmenting LLM inputs with predicted mortality risk and SHAP values improved clinical response quality, as assessed by the IMPACT framework. We further compared GPT-4o with seven contemporary LLMs; all eight models generated clinical support responses that were scored by Claude 3.7 Sonnet to assess performance differences.

**Results:**

Claude 3.7 Sonnet showed excellent agreement with human IMPACT ratings (intraclass correlation coefficient [ICC] 0.979, 95% CI 0.973–0.984) and o3-mini (ICC 0.971, 95% CI 0.964–0.980). In the primary analysis, adding predicted ICU mortality risk and SHAP values significantly increased GPT-4o IMPACT scores across prompting strategies. GPT-5 mini (96.0) and gpt-oss-120B (93.4) outperformed GPT-4o (90.4; both *p* < 0.001) for interpretability and quality.

**Conclusions:**

Combining ML-derived risk, SHAP explanations and LLMs may modestly improve ICU clinical support information, while LLM-based evaluators demonstrated feasibility for scalable evaluation of generated clinical content.

**Supplementary Information:**

The online version contains supplementary material available at 10.1186/s40635-026-00900-w.

## Background

Intensive care unit (ICU) mortality remains high among critically ill patients with multiple comorbidities [1–3], and timely interventions are essential to prevent deterioration and improve clinical outcomes. Machine learning (ML) models have shown improved predictive performance over conventional severity scores in certain clinical settings, with Shapley Additive Explanations (SHAP) providing patient-level interpretability of the model outputs [4, 5]. However, translating these ML-derived predictions and conventional scores into actionable clinical recommendations at the bedside remains challenging [6, 7]. Generative artificial intelligence (AI), especially large language models (LLMs), can synthesize information into comprehensive clinical support outputs and have demonstrated potential for medical question answering and clinical reasoning across diverse specialties [8–10]. These models offer potential to reduce clinician time spent on data interpretation through advanced natural language processing and information synthesis.

Integrating ML with LLMs provides a novel framework for automating actionable insights [11]. After developing ML models for critical care applications [4, 5, 12], our team is establishing a method that first estimates ICU mortality risk using a ML model and then employs a LLM to transform the estimated risk, patient characteristics, and SHAP values into a comprehensive clinical report containing diagnostic interpretations, management recommendations, and follow-up plans. The lack of standardized frameworks for evaluating AI generated clinical support information remains a major barrier [13]. In response, we are developing the IMPACT framework, which includes six domains: Integration, Mastery, Precision, Applicability, Comprehensiveness, and Timeliness. This framework enables systematic assessment of clinical utility, workflow compatibility, interpretive accuracy, contextual relevance, and timeliness of outputs generated from the combined ML and LLM pipeline.

However, two fundamental challenges impede the clinical implementation of this integrated ML-LLM technology. First, the added value of including model-derived information, specifically predicted mortality risk and SHAP values, within LLM inputs has not yet been systematically evaluated. Second, with the rapid emergence of diverse LLM architectures, understanding their relative performance is essential for selecting the most suitable model for specific clinical tasks. In this study, we sought to address these knowledge gaps through a comprehensive investigation. The first goal was to evaluate whether incorporating the ML model’s predicted mortality risk and its SHAP-based explanatory values into the language model input enhances the quality and clinical utility of the generated information. The second goal was to compare the performance of multiple contemporary LLMs, including both proprietary and open-source systems, when applied within this integrated framework.

## Materials and methods

### Data source and ethical approval

This retrospective study analyzed the MIMIC-IV version 3.1 database, comprising 94,458 de-identified ICU admissions (2008–2022, Boston, MA), with data partitioned into training (2008–2016), validation (2017–2019), and testing (2020–2022) cohorts [14–16]. Use of the MIMIC-IV database was approved by the IRBs of MIT (0403000206) and Beth Israel Deaconess Medical Center (2001-P-001699/14), with authorized access granted to a certified team member (CITI No. 32697132, YCY). The complete workflow of dataset allocation for ML model development, prompt optimization, and LLM evaluation is illustrated in Supplementary Fig. 1 (Additional File 1).

### Foundation from previous work

Building upon our previous study [5], we enhanced our approach by developing an XGBoost ML model to predict ICU mortality [17–19]. All features used in the XGBoost model, categorized by domain, are listed in Additional File 1. In the testing dataset, the model achieved an area under the receiver operating characteristic Curve (AUROC) of 0.829 (95% CI 0.816–0.840) and an area under the precision-recall curve (AUPRC) of 0.327 (95% CI 0.300–0.356). The ML model generated predicted mortality risks and SHAP values, which were input into GPT-4o (Microsoft Azure OpenAI, version 2024-11-20) via Python 3.11.11 in Spyder 6.0.6. Three prompting strategies were applied: P1 (simple zero-shot with structured outputs), P2 (advanced multi-level task decomposition with strict formatting and constraints), and P3 (a five-turn conversational extension of P2). GPT-4o then produced five output sections: (A) risk-factor interpretation, (B) recommended examinations, (C) suggested management, (D) follow-up plans, and (E) concise summary. To systematically evaluate the LLM-generated outputs, we used our developed the IMPACT scoring framework, adapted from the validated DISCERN instrument [20], which assesses the quality of written health information. The IMPACT framework comprises six domains: Integration, Mastery, Precision, Applicability, Comprehensiveness, and Timeliness. From this framework, 25 items were selected to evaluate LLM-generated clinical responses, each rated from 0 (poor) to 4 (excellent), yielding a total score range of 0–100. The IMPACT framework has been validated through a multinational panel consensus process involving 58 panelists from 12 countries (manuscript under review). Thirty ICU admissions were randomly selected from the validation dataset, yielding 90 LLM responses (30 per prompting strategy) evaluated with the IMPACT framework. Six clinicians, including intensivists, residents, and nurses, independently assessed each response in panels of three to ensure balanced judgment. Both human and automated evaluator (o3-mini, Microsoft Azure OpenAI, version 2025-01-31) applied the IMPACT criteria to assess clinical accuracy and relevance, with reliability analyzed using a two-way mixed-effects intraclass correlation coefficient (ICC) [21, 22]. The o3-mini evaluated all 90 responses three time, and mean scores were used for analysis. Inter-rater reliability was high: human-to-human ICC 0.836 (95% CI 0.792 to 0.876) and human mean versus o3-mini mean ICC 0.975 (95% CI 0.969 to 0.982).

### Comparison of evaluation performance between LLMs

To compare evaluation performance across LLMs, Claude 3.7 Sonnet (version 2025-02-19, Anthropic) rescored the previously generated set of 90 GPT-4o responses three times. The mean scores were analyzed and compared with those obtained from human raters and o3-mini. Consistency between human experts and LLM evaluators was then assessed using ICC. Consistency between evaluators was assessed using ICC(3,k) for all three pairwise comparisons: Human vs o3-mini, Human vs Claude 3.7 Sonnet, and o3-mini vs Claude 3.7 Sonnet. Pearson correlation coefficients with linear regression were used to examine the relationship between evaluator scores across sections. The line of identity (*y* = *x*) is shown as a reference for perfect agreement in both ranking and magnitude.

### Primary outcome: effect of ICU mortality risk and SHAP information on LLM responses

**Fig. 1:**
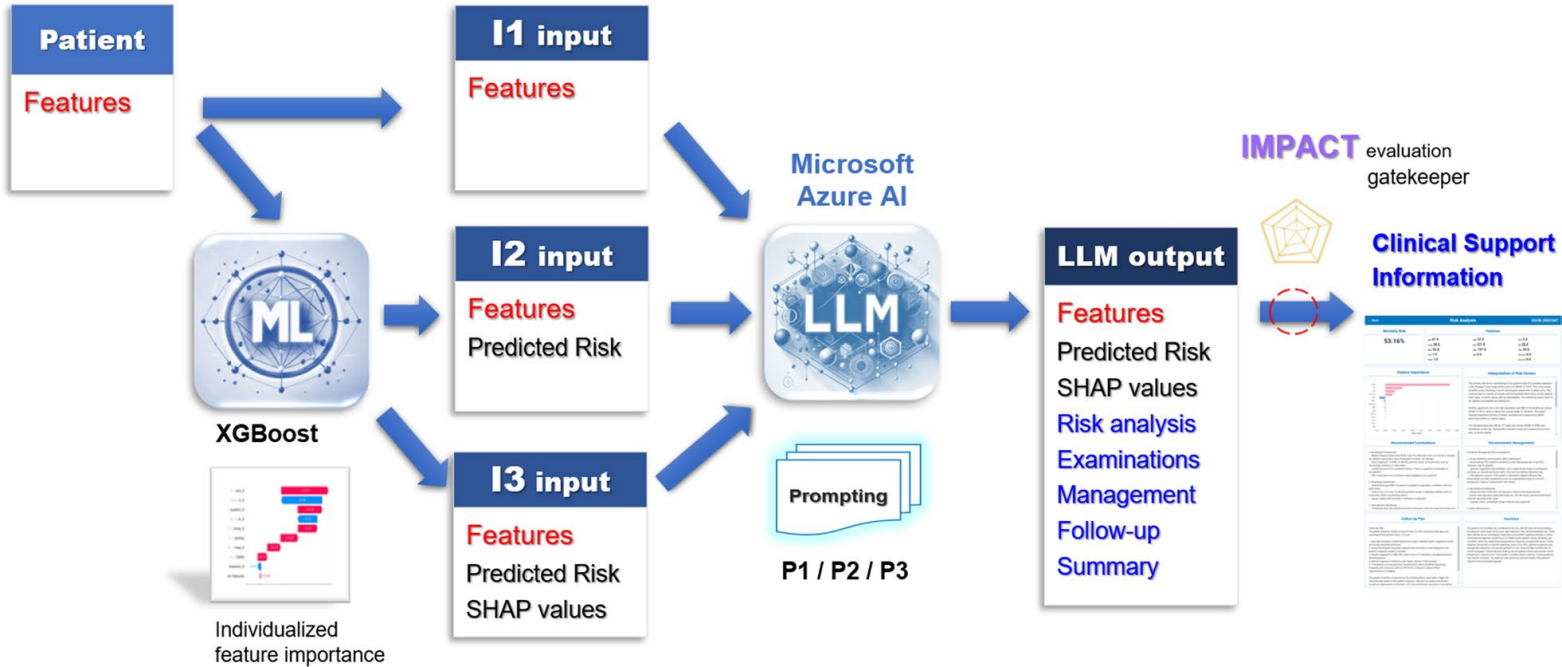
Methodologic workflow of the integrated machine learning and large language model pipeline. Patient features were used to generate predicted mortality risk and SHAP values via XGBoost. These inputs were processed by Microsoft Azure AI LLMs with different prompting strategies to produce structured clinical support information evaluated using the IMPACT framework, which comprises six domains: Integration, Mastery, Precision, Applicability, Comprehensiveness, and Timeliness. LLM, large language model; ML, machine learning. SHAP: Shapley Additive Explanations

The primary analysis examined how predicted ICU mortality risk and SHAP value information influenced the quality of LLM-generated responses. Three input scenarios were evaluated: Input 1 (I1) included only predictor values, Input 2 (I2) added predicted ICU mortality risk, and Input 3 (I3) incorporated predictor values, predicted risk, and individual SHAP values. A total of 120 ICU admissions were randomly selected from the validation dataset, yielding 1,080 responses (120 per combination across nine combinations derived from three prompting strategies and three input scenarios) generated by GPT-4o. These responses were subsequently evaluated using the IMPACT framework by Claude 3.7 Sonnet. The primary outcome compared response quality and clinical reasoning across input scenarios. Comparisons between I1 and I2 assessed the effect of adding predicted ICU mortality risk, while comparisons between I1 and I3 evaluated the combined effect of incorporating both predicted risk and SHAP values. The methodological workflow is illustrated in Fig. 1.

### Exploratory outcomes: performance across multiple LLMs

Exploratory analyses compared GPT-4o performance to seven additional LLMs available through Microsoft Azure AI, includingGPT-4o mini (version 2024-07-08), o3-mini (version 2025-01-31), GPT-5 mini (version 2025.08.07), Grok 3 (version 1), gpt-oss-120B (version 1), Llama 3.3-70B-Instruct (version 5), and Llama 4 Maverick-17B (128E Instruct FP8, version 1). No LLM underwent fine-tuning on ICU-specific content, critical care pathophysiology, or treatment protocols; all models were used in their general-purpose configurations. To ensure consistency, models with adjustable temperature and top_p parameters (GPT-4o mini, Grok 3, Llama 3.3-70B, and Llama 4 Maverick-17B) were configured with a temperature of 0.6 and top_p of 0.9. The reasoning models (o3-mini, GPT-5 mini, and gpt-oss-120B) were used with their default configurations, with reasoning effort set to medium. A total of 360 ICU admissions were randomly selected from the testing dataset, yielding 2,880 responses (360 P3 responses per LLM) generated by the evaluated LLMs. These responses were assessed using the IMPACT framework by Claude 3.7 Sonnet, and the results were compared with those of GPT-4o to determine relative performance across models.

### Statistical analysis

When assessing the effects of ML information on LLM responses, pairwise comparisons of IMPACT scores (I1 vs I2 and I1 vs I3) were conducted using paired *t*-tests. Statistical significance was set at *p* < 0.025 after Bonferroni adjustment for multiple comparisons (*α* = 0.05/2). For comparisons between GP*p*T-4o and the seven other LLMs, paired *t*-tests were conducted. Statistical significance was set at  < 0.007 after Bonferroni adjustment for multiple comparisons (*α* = 0.05/7). Inter-rater agreement was assessed as described above using Pearson correlation coefficients, ICC, and Bland–Altman analysis. Bland–Altman plots display mean bias, 95% limits of agreement with 95% confidence intervals, and proportional bias was tested using linear regression of the difference on the mean. All statistical analyses were performed using IBM SPSS Statistics for Windows, Version 19.0 (IBM Corp., Armonk, NY, USA).

## Results

### Evaluation performance of Claude 3.7 Sonnet compared with human and o3-mini raters

**Fig. 2:**
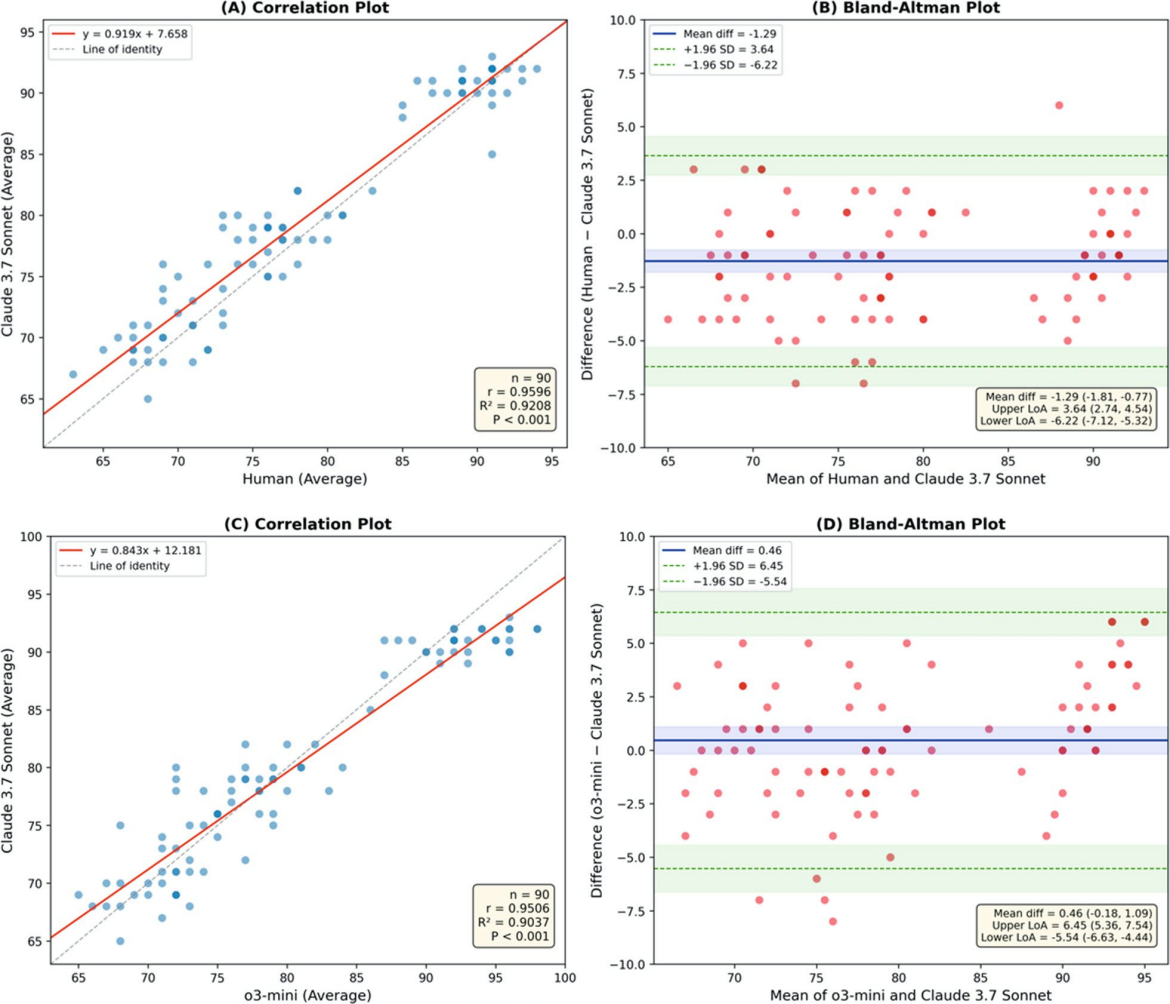
Correlation and Bland–Altman analyses comparing mean IMPACT framework evaluation scores between raters. **A** Correlation plot and **B** Bland–Altman plot comparing Human versus Claude 3.7 Sonnet scores. **C** Correlation plot and **D** Bland–Altman plot comparing o3-mini versus Claude 3.7 Sonnet scores. In the correlation plots, solid red lines represent linear regression and dashed gray lines indicate the line of identity. In the Bland–Altman plots, solid blue lines indicate mean bias, dashed green lines represent 95% limits of agreement, shaded bands denote 95% confidence intervals. Darker spots indicate overlapping data points

**Fig. 3:**
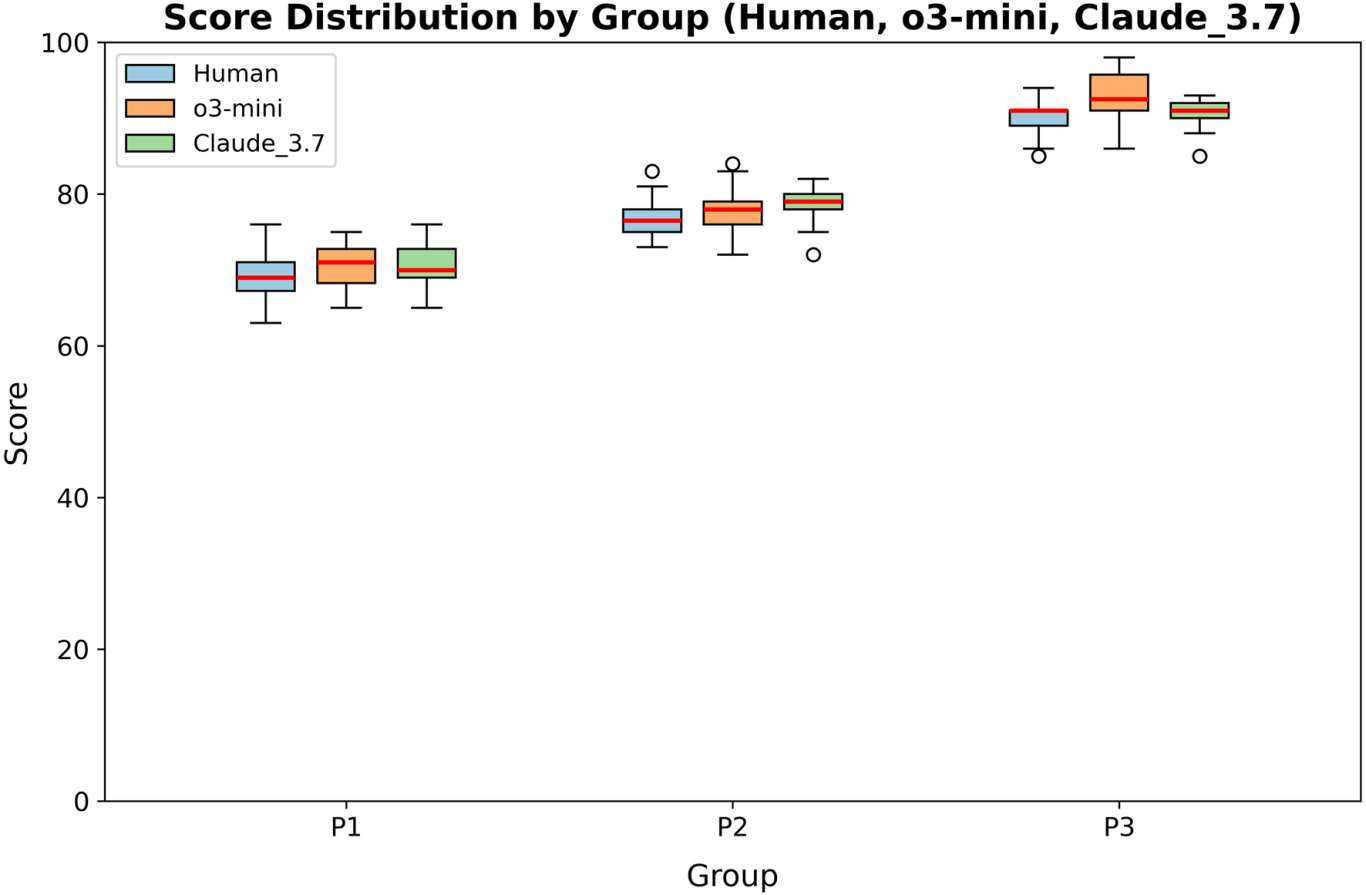
Box plot showing evaluation score distributions from human raters, o3-mini, and Claude 3.7 Sonnet for LLM responses across three prompting strategies. Scores increased progressively from P1 to P3, indicating that richer input information improved evaluation consistency and agreement. Evaluations were performed on GPT-4o–generated responses. LLM, large language model; P1, simple zero-shot structured prompting; P2, advanced zero-shot prompting with explicit guidelines/task decomposition; P3, multi-turn advanced zero-shot prompting with incremental context

Claude 3.7 Sonnet demonstrated excellent intra-rater reliability, with an ICC of 0.994 (95% CI 0.993–0.996). For inter-rater agreement based on mean scores, ICCs were 0.979 (95% CI 0.973–0.984) when compared with human raters and 0.971 (95% CI 0.964–0.980) when compared with o3-mini. Mean difference (Bland–Altman) plots illustrating comparisons between Claude 3.7 Sonnet and human raters, as well as between Claude 3.7 Sonnet and o3-mini, are presented in Fig. 2. The corresponding box plot comparing all three evaluators is shown in Fig. 3. The ICC between human raters and o3-mini was 0.975 (95% CI, 0.966–0.982). Section-level ICC values for all three pairwise comparisons are presented in Supplementary Table 1 (Additional File 1), with ICCs ranging from 0.855 to 0.977 across individual sections. Correlation plots with linear regression analysis for each output section are shown in Supplementary Fig. 2 (Additional File 1), demonstrating strong positive correlations (Pearson *r* = 0.834–0.937) across all pairwise comparisons and sections.

### Primary results: effect of ICU mortality risk and SHAP information on LLM responses

The patient characteristics of the 120 validation cases used in the primary analysis are presented in Supplementary Table 2 (Additional File 1). As shown in Table 1, incorporating ML–derived information significantly improved GPT-4o response quality across all prompting strategies. In P1, mean IMPACT scores increased from 68.4 (I1) to 69.8 (I3) (*p* < 0.001). In P2, scores rose from 72.3 (I1) to 77.6 (I3) (*p* < 0.001). In P3, the highest performance was observed, improving from 88.6 (I1) to 89.8 (I3) (*p* < 0.001). Moreover, adding predicted mortality risk alone (I2) improved performance only in P3, suggesting that conversational prompting better leveraged the added information. Overall, combining predicted risk and SHAP values further enhanced interpretability and comprehensiveness, with the most pronounced improvement observed in P2 and the highest consistency and quality achieved in P3.

**Table 1:** Effects of machine learning-derived information on GPT-4o responses

Information	I1	I2	I3
Mortality risk	–	**V**	**V**
SHAP value	–	–	**V**
P1	68.4 (2.9)	68.8 (2.8)	69.8 (2.7)^#^
P2	72.3 (3.6)	73.1 (3.4)	77.6 (2.6)^#^
P3	88.6 (2.2)	89.6 (1.6)^#^	89.8 (1.8)^#^

*N* = 120 responses per combination generated by GPT-4o (Azure, version 2024-11-20). I1: predictor values only; I2: predictor values plus predicted ICU mortality risk; I3: predictor values, predicted risk, and SHAP values. P1: simple zero-shot with structured outputs; P2: advanced multi-level task decomposition with strict formatting and constraints; P3: five-turn conversational extension of P2. SHAP, Shapley Additive Explanations. Statistical significance was set at *p* < 0.025 after Bonferroni adjustment for multiple comparisons. #*p* < 0.001 compared to I1

### Exploratory results: comparative performance across LLMs

**Fig. 4:**
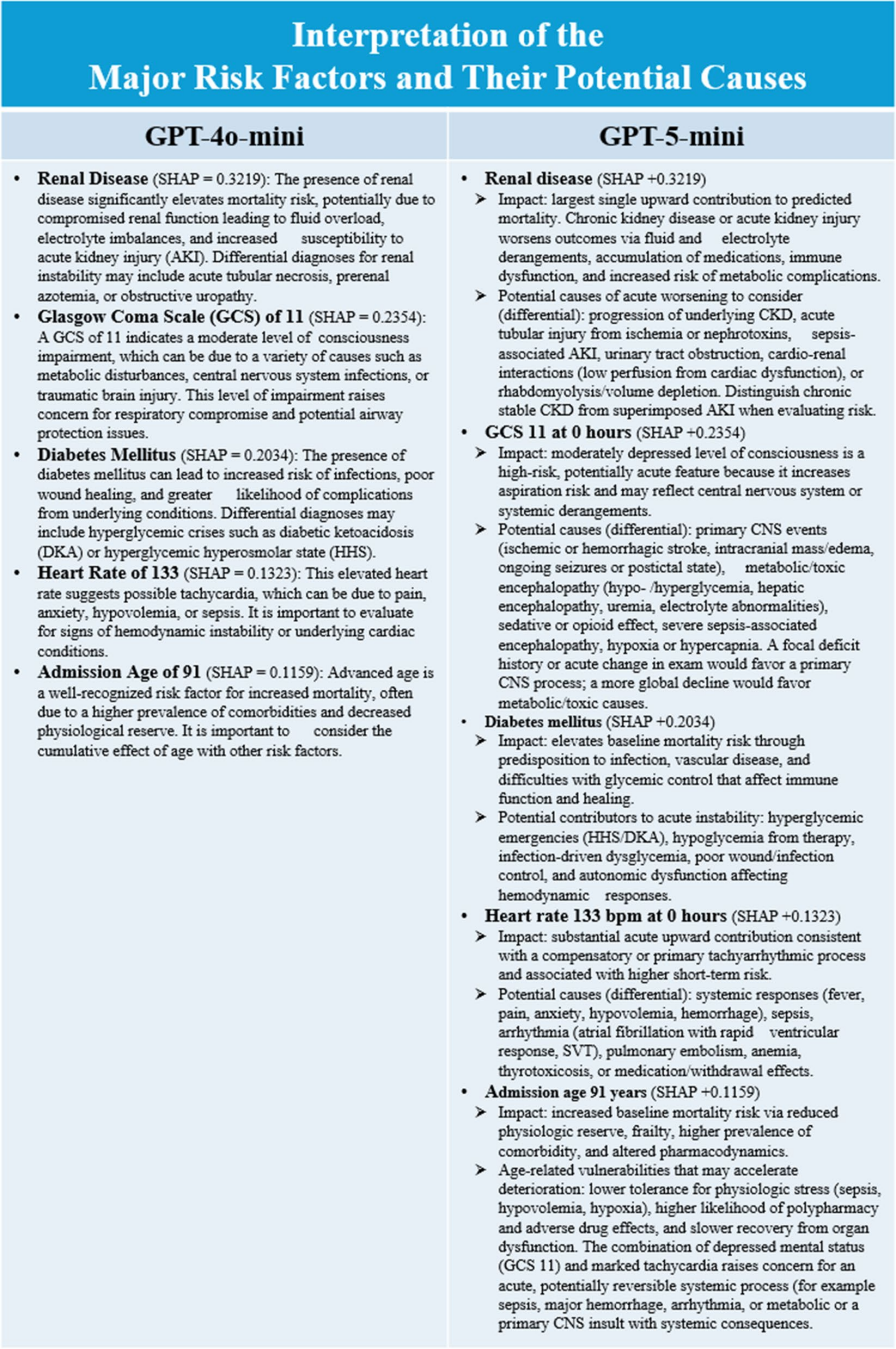
Interpretation of SHAP-Based Major Risk Factors by GPT-4o mini and GPT-5 mini. Representative example of risk-factor interpretation generated by GPT-4o mini (left) and GPT-5 mini (right) for the same ICU patient using SHAP-ranked features. Both models receive identical predictors, predicted mortality risk, and SHAP values. GPT-5 mini provides more structured descriptions of each risk factor’s impact and potential etiologies, including differential diagnoses and pathophysiologic mechanisms, whereas GPT-4o mini yields shorter, template-like explanations. This illustrates qualitative differences in depth, specificity, and clinical usefulness of ML-informed reasoning across LLMs. SHAP: Shapley Additive Explanations

The patient characteristics of the 360 testing cases used in the exploratory analysis are presented in Supplementary Table 3 (Additional File 1). As shown in Table 2, substantial performance variation was observed across LLMs. GPT-5 mini achieved the highest mean IMPACT score of 96.0 (1.0), outperforming GPT-4o (90.4 [1.6], *p* < 0.001), followed by gpt-oss-120B (93.4 [1.3]). GPT-4o remained a balanced model, delivering strong performance with moderate token usage (3.2 k) and computation time (35 s), although its operational cost was relatively high. In contrast, GPT-4o mini, o3-mini, Llama 3.3-70B, and Llama 4-17B showed lower IMPACT scores (78.0–83.7, all *p* < 0.001), indicating weaker integration and clinical reasoning quality. Among proprietary models, Grok-3 achieved a comparable score (88.2 [2.7]) but required the longest generation time (97 s) and highest cost. Open-source gpt-oss-120B demonstrated competitive accuracy with low computational expense, suggesting efficient task suitability. Overall, GPT-5 mini and gpt-oss-120B outperformed others in clinical information generation. In a representative case (Fig. 4), both GPT-4o mini and GPT-5 mini identified the same SHAP-ranked major risk factors but differed in their narrative explanations. GPT-5 mini generated more structured descriptions of each factor’s impact and potential etiologies, including differential diagnoses, whereas GPT-4o mini provided briefer, more template-like summaries.

**Table 2:** IMPACT scores, efficiency, and cost of different large language models

Information	IMPACT score	Output token (k)	Time (s)	Input (USD)	Output (USD)	*P* values
GPT-4o	90.4 (1.6)	3.2 (0.3)	35 (4)	2.50	10.00	–
GPT-4o mini	83.5 (3.6)	2.2 (0.2)	28 (4)	0.15	0.60	< 0.001
GPT-5 mini	96.0 (1.0)	9.4 (0.7)	83 (7)	0.25	2.00	< 0.001
o3-mini	83.7 (2.8)	4.0 (0.4)	46 (9)	1.10	4.40	< 0.001
Grok-3	88.2 (2.7)	2.9 (0.3)	97 (7)	3.00	15.00	< 0.001
gpt-oss-120B	93.4 (1.3)	4.4 (0.5)	26 (3)	0.16	0.60	< 0.001
Llama 3.3-70B	78.3 (2.9)	2.0 (0.2)	42 (3)	0.12	0.30	< 0.001
Llama 4-17B	78.0 (2.5)	1.5 (0.1)	18 (2)	0.15	0.60	< 0.001

*N* = 360 responses per model. LLM responses were evaluated by Claude 3.7 Sonnet (version 2025-02-19, Anthropic) using the IMPACT framework, which comprises six domains: Integration, Mastery, Precision, Applicability, Comprehensiveness, and Timeliness. Paired *t*-tests were used to compare the performance of seven LLMs with GPT-4o. Prices represent cost per 1 M input or output tokens

## Discussion

This study provides two key findings on implementing generative AI for ICU decision support. First, incorporating predicted mortality risk and SHAP values into GPT-4o inputs modestly improved the clinical quality and relevance of generated recommendations, leading to higher IMPACT scores, particularly under structured and conversational prompting strategies. This finding suggests that integrating explicit ML signals into LLM inputs may enhance clinical interpretation beyond summarization of raw patient data. Second, the exploratory comparison across LLMs showed that GPT-5 mini achieved the highest IMPACT score, followed by the open-source gpt-oss-120b, which offered strong performance with lower cost and moderate processing time. These findings underscore the importance of selecting models that balance output quality, computational efficiency, and cost for practical clinical implementation.

The pattern of effects observed across prompting strategies suggests that the benefit of adding mortality risk and SHAP information is real but strongly context dependent [23–25]. Predicted risk and SHAP values consistently increased IMPACT scores, yet the absolute gains were modest, particularly when baseline LLM performance was already high. In the simple zero-shot setting (P1), the prompt led GPT-4o to generate brief, template-like responses, limiting its ability to fully leverage additional structured inputs. In contrast, the structured multi-level task-decomposition prompt (P2) provided explicit sections and detailed instructions, enabling the model to use risk and SHAP cues to deepen diagnostic reasoning, refine risk stratification, and propose more targeted management and follow-up, yielding the largest relative improvement [26–28]. In the five-turn conversational setting (P3), GPT-4o already generated high-quality reports even without SHAP, and the incremental gains from adding SHAP values were small, consistent with a ceiling effect. Importantly, the conversational design enabled regeneration and self-revision across turns, so that the model could infer important patterns from raw predictors and vital signs without always relying on explicit feature attributions. Taken together, these findings indicate that ML-derived risk and SHAP information can enhance clinical coherence and completeness, but their marginal value depends heavily on the quality of the prompt and interaction design. For practical implementation, this suggests two complementary levers to improve ICU decision support: upstream optimization of prediction and explainability, and downstream optimization of prompt structure and conversational workflows.

Our exploratory comparison of eight contemporary LLMs suggests that model selection for ICU decision support involves trade-offs between quality, deployment constraints, and cost. GPT-5 mini achieved the highest IMPACT scores in this study, while the open-source gpt-oss-120b performed competitively [29], indicating that advanced clinical reasoning capabilities may not be limited to proprietary cloud models. GPT-4o offered favorable output quality and latency, though its elevated token cost may restrict routine deployment in resource-limited settings, highlighting the potential value of open-source alternatives for cost-sensitive implementations. Open-source models such as gpt-oss-120b, Llama 3.3-70B, and Llama 4-17B warrant consideration where local deployment or data governance are priorities. Llama 4-17B, optimized for hardware efficiency, achieved performance comparable to Llama 3.3 70B despite having fewer active parameters. In contrast, o3-mini, which is optimised for compact reasoning and tool-oriented workflows, yielded lower IMPACT scores, possibly reflecting its shorter narrative style that may be less comprehensive [30]. These findings suggest that performance on general reasoning benchmarks may not fully predict suitability for structured clinical support tasks and indicate potential value in exploring retrieval-augmented generation and task-specific prompt engineering for both cloud-based and locally deployed models.

This study has several strengths and limitations that inform future work. Strengths include the integration of a rigorously validated XGBoost mortality risk prediction model within a transparent ML–LLM pipeline, and the application of the IMPACT framework, which provides a structured, multidomain evaluation of AI-generated clinical support information. We also integrated multiprofessional human raters with high-agreement LLM evaluators to assess outputs from diverse models in a standardized and consistent manner. Nonetheless, several limitations remain. First, this study should be interpreted as a feasibility investigation. Our findings demonstrate that ML-derived risk estimates and SHAP-based explanations can be incorporated into LLM prompts to generate structured clinical reports. However, routine clinical adoption would require stepwise evaluation, from retrospective accuracy assessment through simulation-based usability testing, including assessment of workflow integration, alert fatigue, and clinician cognitive load, to prospective evaluation of clinician decision-making and patient outcomes. External validation in multicenter cohorts and retrieval-augmented access to current guidelines and local protocols are also needed. Second, the incremental benefit of SHAP-based explanations was modest. When raw clinical values are clearly extreme, their meaning is already apparent, and feature-level attribution adds little. SHAP values might help more when variables fall in borderline ranges where the drivers of risk are less obvious. Additionally, our evaluation was based on a single admission timepoint rather than dynamic clinical decision support. Whether incorporating longitudinal data and serial predictions into LLM prompts improves clinical utility remains to be explored. Third, high agreement between LLM raters may partly reflect shared training data or model biases rather than independent clinical judgment. Future studies should include LLMs from diverse model families and training paradigms to further disentangle true clinical agreement from shared model biases. Fourth, the 0–4 rating scale used in this study, while allowing explicit scoring of absent content (score 0), may compress the range for distinguishing quality among responses that do address the content. Future studies could adopt a 1–5 scale to improve discrimination across the quality spectrum. Fifth, the MIMIC-IV database includes a heterogeneous ICU population, and the effects of ML-derived information on LLM output quality may vary across patient subgroups. Future work should explore subgroup-specific analyses, including clinical subphenotyping approaches [31]. Sixth, using Claude 3.7 Sonnet as the primary evaluator may introduce bias favoring responses matching its generative patterns, although we selected an evaluator from a different model family than the majority of evaluated LLMs to mitigate this risk. Moreover, domain-specific fine-tuning on critical care data could potentially enable smaller, less computationally demanding models to achieve comparable performance, and this represents an important direction for future investigation.

## Conclusions

Integrating ML-derived mortality risk, SHAP explanations, and carefully designed prompting with LLMs modestly improved the coherence and completeness of ICU clinical support information in this conceptual study. Combined with structured evaluation using the IMPACT framework, these findings support a cautious, stepwise approach and prospective validation of ML–LLM pipelines before considering their routine use in critical care practice.

## Supplementary Information

Additional file1 (DOCX 516 kb)

## Data Availability

The data underpinning this study are accessible through the MIMIC-IV database via PhysioNet, subject to credentialing, completion of required training, and execution of a data-use agreement. Additional supporting data that enable replication of the study results are available from the corresponding authors upon reasonable request.

## Abbreviations

AI: Artificial Intelligence
CI: Confidence Interval
ICC: Intraclass Correlation Coefficient
ICU: Intensive Care Unit
IMPACT: Integration, Mastery, Precision, Applicability, Comprehensiveness, and Timeliness
LLM: Large Language Model
MIMIC-IV: Medical Information Mart for Intensive Care IV
ML: Machine Learning
SHAP: Shapley Additive Explanations
