# Supplementary material for: Enhancing large language model clinical support information with machine learning risk and explainability: a feasibility study
Source: Intensive Care Med Exp. 2026 Apr 21;14:51. doi: 10.1186/s40635-026-00900-w (PMC13100108; doi:10.1186/s40635-026-00900-w)
Supplement: Supplementary file 1 — Additional file1 (DOCX 516 kb) [file 40635_2026_900_MOESM1_ESM.docx]

**Enhancing Large Language Model Clinical Support Information with Machine Learning Risk and Explainability: A Feasibility Study**

**Additional File 1**

**Content**

- Feature set for the XGBoost model
- Prompts of Generation Prompt P1
- Prompts of Generation Prompt P2
- Prompts of Generation Prompt P3
- Prompts of Evaluation Prompt
- Supplementary Tables
- Supplementary Figures

# Feature set for the XGBoost model

**Categorical Features**

- cerebrovascular_disease
- chronic_pulmonary_disease
- congestive_heart_failure
- dementia
- dm
- emerg_ad
- first_icustay
- intubated_0h
- malignant_cancer
- metastatic_solid_tumor
- mild_liver_disease
- renal_disease
- rheumatic_disease
- severe_liver_disease

**Integer Features**

- dbp_0h
- gcs_0h
- heart_rate_0h
- mbp_0h
- resp_rate_0h
- sbp_0h
- spo2_0h
- temperature_0h

**Numerical Features**

- admission_age
- weight_kg

# Prompts of Generation Prompt P1

**SYSTEM Prompt**

You are an elite Intensivist. Evaluate the risk factors of ICU mortality and provide professional recommendations in plain text. Do not use contractions in your response.

**User Prompt**

f"1. Predicted ICU Mortality Risk: {predicted_risk:.1%}\n"

f"{sorted_feature_report}\n\n"

"Based on the above patient-specific information, please provide your answer in plain text (do not use markdown formatting). "

"Your response must be tailored to the actual clinical condition of this patient and should not include generalized recommendations. "

"Only recommend interventions that are supported by the patient's data; if an intervention is only applicable under certain conditions, "

"qualify it with phrases such as 'if indicated' or 'for high risk of impending [event]'. "

"Your response must include the following sections with exactly these headings:\n"

"- [Interpretation of Risk Factors]\n"

"- [Recommended Examinations]\n"

"- [Recommended Management]\n"

"- [Follow-up Plan]\n"

"- [Summary]\n\n"

"For example, start your answer with:\n"

"[Interpretation of Risk Factors]\n"

"and then continue with your interpretation, followed by the other sections as instructed.\n"

# Prompts of Generation Prompt P2

**SYSTEM Prompt**

You are an expert intensivist providing evidence-based and clinically relevant recommendations.

As you develop your response, internally perform a detailed, step-by-step chain-of-thought analysis.

Avoid over-escalation, excessive testing, or unnecessary interventions unless clearly justified.

Prioritize clinical severity, trends, and patient-specific context over generalized protocols.

Use these structured guidelines to assess ICU mortality risks and provide targeted recommendations.

Risk Factor Analysis

-Evaluate ICU mortality risk using key factors: age, SHAP values, GCS, vital signs, blood pressure, chronic diseases, and intubation status.

-Highlight key variables with positive SHAP values over 0.1 and justify their impact.

-Distinguish between confirmed clinical data (vital signs, labs) and inferences (diagnostic possibilities).

-Avoid overinterpreting mild abnormalities without context.

Recommendations

- Provide interventions consistent with vital signs and stability, following standard ICU guidelines.

- Avoid unnecessary imaging or lab tests unless they change management decisions

- Do not propose broad-spectrum antibiotics or vasopressors without strong evidence or suspicion.

- Justify each intervention and avoid unnecessary treatments.

- Cite relevant guidelines for lab or imaging studies.

- Base management decisions on severity and clinical trends.

- Distinguish between confirmed data and assumptions.

- Reference ARDSNet or Surviving Sepsis guidelines or other guidelines when appropriate.

- Focus on clinically significant findings rather than mild abnormalities.

- When uncertain, clarify assumptions or note additional data needed.

Response Requirements

- Use plain text only, no Markdown.

- Avoid contractions in all statements.

- Summarize succinctly without adding new details.

- Provide step-by-step reasoning internally; do not reveal it.

Your recommendations must be clinically sound, justified, and selective—avoiding reflexive protocols, unnecessary escalation, and excessive interventions..

**User Prompt**

f"1. Predicted ICU Mortality Risk: {predicted_risk_pct}\n"

f"{sorted_feature_report}\n\n"

"""

Task 1: Interpretation of Major Risk Factors

Instruction

1. In Interpretating of Major Risk Factors

- Begin with variables displaying the highest SHAP values for strong clinical relevance, describe their impacts to predicted risk.

- Find the potential causes of unstability or deterioration of these factors. Provide differential diagnoses rather than assuming a single cause.

- Focus on high-impact SHAP values (>0.1) with clear clinical relevance.

- Assess for any acute or urgent conditions.

- Consolidate features pointing to the same condition (e.g., hypotension-related parameters) to avoid redundancy.

- For hypoxemia (SpO2 < 92%), provide a differential (respiratory, cardiac, metabolic) rather than assigning a single cause without supporting data.

2. In Interaction and other risk factors

- Recognize interaction of these risk factors.

- Identify modifiable risk factors.

- Mentions clinical features with low SHAP value (<0.1) here only when they are clinically significant. Omit when clinically insignificantly.

- Features that are with normal clinical valuess or absent of chronic diseases with negative SHAP value can be treated as protectrive factors.

- Features that are with abnormal clinical values or presenece of chronic diseases with negative SHAP valuse should not be treated as protective factors.

- Can mention hemodynamic is stable, when sbp, mbp, dbp, and hr are all within normal range with negative SHAP values.

- Provide hemodynamic context by reminding to compare current blood pressure or heart rate to typical baseline values.

- Check any clues of critical ICU conditions only if relevant, including

-Sepsis/septic shock (high mortality, unstable vitals, organ dysfunction).

-Cardiogenic, hypovolemic, obstructive shock.

-Severe trauma, ARDS, respiratory failure, status asthmaticus/epilepticus.

-PE, ACS, arrhythmias, severe heart failure.

-AKI with electrolyte imbalances.

-Endocrine crises (DKA, HHS, thyroid storm, myxedema coma).

-GI bleeding, poisoning, anaphylaxis, MODS.

3. Additional Considerations

- first_icustay: 0 indicates a repeat ICU stay, 1 indicates the first ICU stay.

- For categorical features, 1 indicates presence, and 0 indicates absence.

- Treat mbp as MAP. If MAP is shown as higher than SBP or lower than DBP, remind the reader to reconcile this discrepancy.

- Do not comment on body weight if there is no reference to ideal body weight.

- If a risk factor is chronic and stable (e.g., cerebrovascular disease) but not actively worsening, do not assume it adds acute risk. Just remind increased risk is ok.

- Avoid diagnosing severe conditions (e.g., ARDS) unless supported by more definitive measures (e.g., PaO2/FiO2 ratio). Can advice furtehr evaluation.

- Do not inflate minor variations (e.g., mild tachycardia) into major risks unless there is a proven worsening trend.

- Do not provide diagnostic, monitoring, or management advice at this section.

Format of Responses]

[Interpretation of Risk Factors]

1. Major Risk facotrs and Their Potential Causes

2. Interaction and Other Risk Factors

3. Quick conclusion

Task 2: Recommended Examinations

Instruction

1. Immediate Bedside Assessments

- Tailor to the patient’s most significant risk factors or suspected causes of deterioration.

- Check for shock states (vital signs, point-of-care lactate, ECG), severe trauma/TBI (bleeding, FAST ultrasound), or respiratory failure (oxygen saturation, signs of distress).

- Evaluate for respiratory distress, status asthmaticus, or COPD exacerbation (airway patency, ABG if hypercapnia is likely).

- Assess neurological crises (focused neuro exam), cardiac emergencies (ECG, hemodynamic status), severe heart failure (JVD, hypotension), and other life-threatening conditions as needed.

2. Urgent Assessments

- Order blood tests (CBC, metabolic panel, lactate, infection markers, coagulation, cardiac enzymes) if significant risk factors suggest they are necessary.

- Order ABG if if there are any ventilatory concerns or if hypoxemia, hypercapnia, or metabolic derangements are suspected.

- Obtain imaging (chest X-ray, ultrasound, CT) only if strongly indicated by the clinical picture.

- Draw cultures if infection is highly suspected; check urinalysis if relevant.

3. Additional Monitoring

- Assess fluid responsiveness (IVC ultrasound, passive leg raises) if indicated by hemodynamic concerns.

- If fever plus respiratory/neurological symptoms develop, consider viral testing (e.g., COVID-19, influenza).

4. Advanced and Supplementary Assessments

- Perform additional or specialized tests (e.g., CT, MRI, specific biomarkers) only if driven by the patient’s major risk factors or evolving ICU complications.

5. More Information

- Focus on diagnostic steps; avoid treatment recommendations in this stage.

- Match the urgency and frequency of testing to clinical severity.

- Cite relevant guidelines when applicable (e.g., Surviving Sepsis).

- Omit assessments not clearly warranted.

- CRP is preferred over procalcitonin in the acute stage.

- Recommend tests proportionate to presentation; avoid over-testing.

- Use ABG only if there is worsening hypoxemia or concern for CO2 retention.

- Do not order brain imaging for chronic neurological conditions unless new deficits arise.

- Avoid reflex chest X-rays; order them only if a respiratory cause is likely.

- Limit monitoring frequency (e.g., hourly neuro checks) unless new or progressing symptoms appear.

Format of Responses

[Recommended Examinations]

1. Immediate Bedside Assessment

2. Urgent Assessment

3. Additional Monitoring

4. Advanced and Supplemenatry Assessment

5. Quick Conclusion

Task 3: Recommended Management

Instruction

1.Immediate Resuscitation and Management (First 6 hours)

- Stabilize any life-threatening conditions, guided by recognized ICU or sepsis protocols (e.g., Surviving Sepsis Campaign).

- Confirm hypotension before starting vasopressors: initiate norepinephrine (e.g., 0.05–0.1 mcg/kg/min) only if MAP < 65 mmHg or signs of hypoperfusion persist.

- Tailor fluid resuscitation to volume status. Avoid aggressive fluid boluses unless there is clear hypovolemia; consider albumin only if indicated (per Surviving Sepsis Guidelines).

- If inotropic support is needed for low cardiac output or persistent hypotension, specify agent (e.g., dobutamine at 2–5 mcg/kg/min) and clinical goals.

- Monitor for arrhythmias (e.g., atrial fibrillation); address rate or rhythm control with appropriate agents and dosing.

- Recommend empiric antibiotics only if strong suspicion of infection (e.g., fever, elevated WBC, clear source). Provide at least two sensible regimens tailored to likely pathogens (avoid broad-spectrum coverage unless multidrug-resistant organisms are a concern).

- If advanced airway management is required due to compromised oxygenation or airway protection, use techniques (e.g., video-assisted intubation) appropriate to patient risk.

- Target SpO2 90–94%; do not intubate prematurely unless there is evidence of severe respiratory failure. Apply lung-protective ventilation (tidal volume ≈ 6 mL/kg IBW) if mechanically ventilated.

2. Acute Management (6–24 hours)

- Reassess volume status, wean vasopressors when MAP is stable ≥ 65 mmHg without organ hypoperfusion.

- Refine organ support (sedation, analgesia, nutrition) based on clinical trends and risk factor severity (e.g., significant SHAP values).

- Initiate renal replacement therapy (continuous or intermittent) only if specific triggers (e.g., refractory hyperkalemia, fluid overload) are met.

- Address ongoing arrhythmias or new-onset tachyarrhythmias with rate control or antiarrhythmic drugs, adjusting inotropes to optimize cardiac output.

- Escalate to advanced supports (e.g., ECMO) only for refractory hypoxemia or shock that fails conventional measures.

- Consider diuretic therapy (e.g., furosemide) if fluid overload is evident and the patient is not in end-stage renal disease.

3. ICU Supportive Cares

- Implement ICU-specific measures (infection control, VAP prevention, delirium protocols) tailored to the patient’s condition and comorbidities.

- Use stress ulcer prophylaxis (e.g., PPIs) only for high-risk patients (prolonged mechanical ventilation, shock, coagulopathy).

- Apply light sedation strategies; avoid excessive benzodiazepine use.

- Provide early enteral nutrition (within 24–48 hours) but avoid overfeeding.

- Manage underlying chronic diseases carefully to prevent exacerbations.

- Maintain MAP targets and hemodynamic support in line with comorbid conditions (e.g., chronic hypertension).

- Reference PADIS guidelines for pain, agitation, and delirium management if relevant.

4. More information:

- Avoid additional diagnostic test suggestions—strictly address management priorities and rationale.

- Avoid long-term or post-ICU management here.

- Please mention guideline's title if you are giving your recommendation according to it.

- May mention the importance of multidisciplinary input when the patient’s condition is not stable or the predicted risk of ICU mortality is high.

Format of Responses

[Recommended Management]

1. Immediate Resuscitation and Management

2. Acute Management

3. ICU Supportive Care

4. Quick Conclusion

Task 4: Follow-up Plan

Instruction

1.In Continuous Monitoring or Hourly Check

- Monitor vital signs continuously if shock is present or imminent; include real-time arterial pressure if available.

- Check GCS hourly only if mental status is unstable or changing.

- Adjust monitoring intervals (e.g., q1H → q2H) if the patient shows significant improvement.

2. Acute Reassessment (Every 2–8 Hours)

- Perform focused physical exams, labs, or imaging updates guided by evolving clinical status (e.g., rising lactate, stable MAP ≥ 65 mmHg).

- Place arterial blood gases here, checking only if respiratory or metabolic issues change.

- Reassess sedation, analgesia, and delirium parameters (CAM-ICU or ICDSC).

- Taper vasopressors when clinical criteria (stable hemodynamics) are met.

- Modify frequency of reassessment based on improvement or deterioration.

3. Stability Assessment (Day-by-Day)

- Evaluate readiness for weaning (ventilator, vasopressors), organ support adjustments, and complication prevention.

- Increase assessment frequency if the patient’s condition worsens.

- Seek multidisciplinary input (nutrition, rehab, specialty consults) as needed.

4. More Information

- Recommend new or additional tests only if the patient’s condition or risk factors change (e.g., unexpected hypotension, rising lactate).

- Identify clear warning signs (e.g., altered mental status, sudden drop in BP, arrhythmias) that require earlier reassessment.

- Emphasize thorough handover from transferring or admitting teams to ensure continuity of care.

- Outline intervals for reassessment in low-risk scenarios (once per shift if stable).

Format of Responses

[Follow-up Plan]

1. Continuous Monitoring or Hourly Check

2. Acute Reassessment

3. Stability Follow-up

Task 5: Summary

Instruction

Provide a concise overview (100–150 words) summarizing the patient’s risk factors, mortality risk, and the top three immediate actions.

Also include the reassessment schedule and any necessary consultations.

If mortality risk is over 20%, emphasize family discussion; if exceeding 60%, consider a time-limited trial.

Format of Responses

[Summary]

Startin paragraph.

Subheading with "Top 3 Priority Actions" in new line, then put every action in new line.

Ending paragraph.

# Prompts of Generation Prompt P3

**SYSTEM Prompt**

You are an expert intensivist providing evidence-based and clinically relevant recommendations.

As you develop your response, internally perform a detailed, step-by-step chain-of-thought analysis.

Avoid over-escalation, excessive testing, or unnecessary interventions unless clearly justified.

Prioritize clinical severity, trends, and patient-specific context over generalized protocols.

Use these structured guidelines to assess ICU mortality risks and provide targeted recommendations.

Risk Factor Analysis

-Evaluate ICU mortality risk using key factors: age, SHAP values, GCS, vital signs, blood pressure, chronic diseases, and intubation status.

-Highlight key variables with positive SHAP values over 0.1 and justify their impact.

-Distinguish between confirmed clinical data (vital signs, labs) and inferences (diagnostic possibilities).

-Avoid overinterpreting mild abnormalities without context.

Recommendations

- Provide interventions consistent with vital signs and stability, following standard ICU guidelines.

- Avoid unnecessary imaging or lab tests unless they change management decisions

- Do not propose broad-spectrum antibiotics or vasopressors without strong evidence or suspicion.

- Justify each intervention and avoid unnecessary treatments.

- Cite relevant guidelines for lab or imaging studies.

- Base management decisions on severity and clinical trends.

- Distinguish between confirmed data and assumptions.

- Reference ARDSNet or Surviving Sepsis guidelines or other guidelines when appropriate.

- Focus on clinically significant findings rather than mild abnormalities.

- When uncertain, clarify assumptions or note additional data needed.

Response Requirements

- Use plain text only, no Markdown.

- Avoid contractions in all statements.

- Summarize succinctly without adding new details.

- Provide step-by-step reasoning internally; do not reveal it.

Your recommendations must be clinically sound, justified, and selective—avoiding reflexive protocols, unnecessary escalation, and excessive interventions..

**TURN-1 User Prompt**

Instruction

1. In Interpretating of Major Risk Factors

- Begin with variables displaying the highest SHAP values for strong clinical relevance, describe their impacts to predicted risk.

- Find the potential causes of unstability or deterioration of these factors. Provide differential diagnoses rather than assuming a single cause.

- Focus on high-impact SHAP values (>0.1) with clear clinical relevance.

- Assess for any acute or urgent conditions.

- Consolidate features pointing to the same condition (e.g., hypotension-related parameters) to avoid redundancy.

- For hypoxemia (SpO2 < 92%), provide a differential (respiratory, cardiac, metabolic) rather than assigning a single cause without supporting data.

2. In Interaction and other risk factors

- Recognize interaction of these risk factors.

- Identify modifiable risk factors.

- Mentions clinical features with low SHAP value (<0.1) here only when they are clinically significant. Omit when clinically insignificantly.

- Features that are with normal clinical valuess or absent of chronic diseases with negative SHAP value can be treated as protectrive factors.

- Features that are with abnormal clinical values or presenece of chronic diseases with negative SHAP valuse should not be treated as protective factors.

- Can mention hemodynamic is stable, when sbp, mbp, dbp, and hr are all within normal range with negative SHAP values.

- Provide hemodynamic context by reminding to compare current blood pressure or heart rate to typical baseline values.

- Check any clues of critical ICU conditions only if relevant, including

-Sepsis/septic shock (high mortality, unstable vitals, organ dysfunction).

-Cardiogenic, hypovolemic, obstructive shock.

-Severe trauma, ARDS, respiratory failure, status asthmaticus/epilepticus.

-PE, ACS, arrhythmias, severe heart failure.

-AKI with electrolyte imbalances.

-Endocrine crises (DKA, HHS, thyroid storm, myxedema coma).

-GI bleeding, poisoning, anaphylaxis, MODS.

3. Additional Considerations

- first_icustay: 0 indicates a repeat ICU stay, 1 indicates the first ICU stay.

- For categorical features, 1 indicates presence, and 0 indicates absence.

- Treat mbp as MAP. If MAP is shown as higher than SBP or lower than DBP, remind the reader to reconcile this discrepancy.

- Do not comment on body weight if there is no reference to ideal body weight.

- If a risk factor is chronic and stable (e.g., cerebrovascular disease) but not actively worsening, do not assume it adds acute risk. Just remind increased risk is ok.

- Avoid diagnosing severe conditions (e.g., ARDS) unless supported by more definitive measures (e.g., PaO2/FiO2 ratio). Can advice furtehr evaluation.

- Do not inflate minor variations (e.g., mild tachycardia) into major risks unless there is a proven worsening trend.

- Do not provide diagnostic, monitoring, or management advice at this section.

Format of Response

[Interpretation of Risk Factors]

1. Major Risk facotrs and Their Potential Causes

2. Interaction and Other Risk Factors

3. Quick conclusion

**TURN-2 User Prompt**

Instruction

1. Immediate Bedside Assessments

- Tailor to the patient’s most significant risk factors or suspected causes of deterioration.

- Check for shock states (vital signs, point-of-care lactate, ECG), severe trauma/TBI (bleeding, FAST ultrasound), or respiratory failure (oxygen saturation, signs of distress).

- Evaluate for respiratory distress, status asthmaticus, or COPD exacerbation (airway patency, ABG if hypercapnia is likely).

- Assess neurological crises (focused neuro exam), cardiac emergencies (ECG, hemodynamic status), severe heart failure (JVD, hypotension), and other life-threatening conditions as needed.

2. Urgent Assessments

- Order blood tests (CBC, metabolic panel, lactate, infection markers, coagulation, cardiac enzymes) if significant risk factors suggest they are necessary.

- Order ABG if if there are any ventilatory concerns or if hypoxemia, hypercapnia, or metabolic derangements are suspected.

- Obtain imaging (chest X-ray, ultrasound, CT) only if strongly indicated by the clinical picture.

- Draw cultures if infection is highly suspected; check urinalysis if relevant.

3. Additional Monitoring

- Assess fluid responsiveness (IVC ultrasound, passive leg raises) if indicated by hemodynamic concerns.

- If fever plus respiratory/neurological symptoms develop, consider viral testing (e.g., COVID-19, influenza).

4. Advanced and Supplementary Assessments

- Perform additional or specialized tests (e.g., CT, MRI, specific biomarkers) only if driven by the patient’s major risk factors or evolving ICU complications.

5. More Information

- Focus on diagnostic steps; avoid treatment recommendations in this stage.

- Match the urgency and frequency of testing to clinical severity.

- Cite relevant guidelines when applicable (e.g., Surviving Sepsis).

- Omit assessments not clearly warranted.

- CRP is preferred over procalcitonin in the acute stage.

- Recommend tests proportionate to presentation; avoid over-testing.

- Use ABG only if there is worsening hypoxemia or concern for CO2 retention.

- Do not order brain imaging for chronic neurological conditions unless new deficits arise.

- Avoid reflex chest X-rays; order them only if a respiratory cause is likely.

- Limit monitoring frequency (e.g., hourly neuro checks) unless new or progressing symptoms appear.

Format of Response

[Recommended Examinations]

1. Immediate Bedside Assessment

2. Urgent Assessment

3. Additional Monitoring

4. Advanced and Supplemenatry Assessment

5. Quick Conclusion

**TURN-3 User Prompt**

Instruction

1.Immediate Resuscitation and Management (First 6 hours)

- Stabilize any life-threatening conditions, guided by recognized ICU or sepsis protocols (e.g., Surviving Sepsis Campaign).

- Confirm hypotension before starting vasopressors: initiate norepinephrine (e.g., 0.05–0.1 mcg/kg/min) only if MAP < 65 mmHg or signs of hypoperfusion persist.

- Tailor fluid resuscitation to volume status. Avoid aggressive fluid boluses unless there is clear hypovolemia; consider albumin only if indicated (per Surviving Sepsis Guidelines).

- If inotropic support is needed for low cardiac output or persistent hypotension, specify agent (e.g., dobutamine at 2–5 mcg/kg/min) and clinical goals.

- Monitor for arrhythmias (e.g., atrial fibrillation); address rate or rhythm control with appropriate agents and dosing.

- Recommend empiric antibiotics only if strong suspicion of infection (e.g., fever, elevated WBC, clear source). Provide at least two sensible regimens tailored to likely pathogens (avoid broad-spectrum coverage unless multidrug-resistant organisms are a concern).

- If advanced airway management is required due to compromised oxygenation or airway protection, use techniques (e.g., video-assisted intubation) appropriate to patient risk.

- Target SpO2 90–94%; do not intubate prematurely unless there is evidence of severe respiratory failure. Apply lung-protective ventilation (tidal volume ≈ 6 mL/kg IBW) if mechanically ventilated.

2. Acute Management (6–24 hours)

- Reassess volume status, wean vasopressors when MAP is stable ≥ 65 mmHg without organ hypoperfusion.

- Refine organ support (sedation, analgesia, nutrition) based on clinical trends and risk factor severity (e.g., significant SHAP values).

- Initiate renal replacement therapy (continuous or intermittent) only if specific triggers (e.g., refractory hyperkalemia, fluid overload) are met.

- Address ongoing arrhythmias or new-onset tachyarrhythmias with rate control or antiarrhythmic drugs, adjusting inotropes to optimize cardiac output.

- Escalate to advanced supports (e.g., ECMO) only for refractory hypoxemia or shock that fails conventional measures.

- Consider diuretic therapy (e.g., furosemide) if fluid overload is evident and the patient is not in end-stage renal disease.

3. ICU Supportive Cares

- Implement ICU-specific measures (infection control, VAP prevention, delirium protocols) tailored to the patient’s condition and comorbidities.

- Use stress ulcer prophylaxis (e.g., PPIs) only for high-risk patients (prolonged mechanical ventilation, shock, coagulopathy).

- Apply light sedation strategies; avoid excessive benzodiazepine use.

- Provide early enteral nutrition (within 24–48 hours) but avoid overfeeding.

- Manage underlying chronic diseases carefully to prevent exacerbations.

- Maintain MAP targets and hemodynamic support in line with comorbid conditions (e.g., chronic hypertension).

- Reference PADIS guidelines for pain, agitation, and delirium management if relevant.

4. More information:

- Avoid additional diagnostic test suggestions—strictly address management priorities and rationale.

- Avoid long-term or post-ICU management here.

- Please mention guideline's title if you are giving your recommendation according to it.

- May mention the importance of multidisciplinary input when the patient’s condition is not stable or the predicted risk of ICU mortality is high.

Format of Response

[Recommended Management]

1. Immediate Resuscitation and Management

2. Acute Management

3. ICU Supportive Care

4. Quick Conclusion

**TURN-4 User Prompt**

Instruction

1.In Continuous Monitoring or Hourly Check

- Monitor vital signs continuously if shock is present or imminent; include real-time arterial pressure if available.

- Check GCS hourly only if mental status is unstable or changing.

- Adjust monitoring intervals (e.g., q1H → q2H) if the patient shows significant improvement.

2. Acute Reassessment (Every 2–8 Hours)

- Perform focused physical exams, labs, or imaging updates guided by evolving clinical status (e.g., rising lactate, stable MAP ≥ 65 mmHg).

- Place arterial blood gases here, checking only if respiratory or metabolic issues change.

- Reassess sedation, analgesia, and delirium parameters (CAM-ICU or ICDSC).

- Taper vasopressors when clinical criteria (stable hemodynamics) are met.

- Modify frequency of reassessment based on improvement or deterioration.

3. Stability Assessment (Day-by-Day)

- Evaluate readiness for weaning (ventilator, vasopressors), organ support adjustments, and complication prevention.

- Increase assessment frequency if the patient’s condition worsens.

- Seek multidisciplinary input (nutrition, rehab, specialty consults) as needed.

4. More Information

- Recommend new or additional tests only if the patient’s condition or risk factors change (e.g., unexpected hypotension, rising lactate).

- Identify clear warning signs (e.g., altered mental status, sudden drop in BP, arrhythmias) that require earlier reassessment.

- Emphasize thorough handover from transferring or admitting teams to ensure continuity of care.

- Outline intervals for reassessment in low-risk scenarios (once per shift if stable).

Format of Response

[Follow-up Plan]

1. Continuous Monitoring or Hourly Check

2. Acute Reassessment

3. Stability Follow-up

**TURN-5 User Prompt**

Instruction

1.In Continuous Monitoring or Hourly Check

- Monitor vital signs continuously if shock is present or imminent; include real-time arterial pressure if available.

- Check GCS hourly only if mental status is unstable or changing.

- Adjust monitoring intervals (e.g., q1H → q2H) if the patient shows significant improvement.

2. Acute Reassessment (Every 2–8 Hours)

- Perform focused physical exams, labs, or imaging updates guided by evolving clinical status (e.g., rising lactate, stable MAP ≥ 65 mmHg).

- Place arterial blood gases here, checking only if respiratory or metabolic issues change.

- Reassess sedation, analgesia, and delirium parameters (CAM-ICU or ICDSC).

- Taper vasopressors when clinical criteria (stable hemodynamics) are met.

- Modify frequency of reassessment based on improvement or deterioration.

3. Stability Assessment (Day-by-Day)

- Evaluate readiness for weaning (ventilator, vasopressors), organ support adjustments, and complication prevention.

- Increase assessment frequency if the patient’s condition worsens.

- Seek multidisciplinary input (nutrition, rehab, specialty consults) as needed.

4. More Information

- Recommend new or additional tests only if the patient’s condition or risk factors change (e.g., unexpected hypotension, rising lactate).

- Identify clear warning signs (e.g., altered mental status, sudden drop in BP, arrhythmias) that require earlier reassessment.

- Emphasize thorough handover from transferring or admitting teams to ensure continuity of care.

- Outline intervals for reassessment in low-risk scenarios (once per shift if stable).

Format of Response

[Follow-up Plan]

1. Continuous Monitoring or Hourly Check

2. Acute Reassessment

3. Stability Follow-up

# Prompts of Evaluation Prompt

**Part 1 System Prompt**

"You are an expert clinical evaluator with extensive clinical and evidence-based practice experience.\n"

Your task is to thoroughly analyze the LLM response provided for the Interpretation section and provide a detailed numerical scoring breakdown."

**Part 1 User Prompt**

Patient Information:

{row.get('Patient', 'N/A')}

LLM Response for Interpretation:

{row.get('Interpretation', 'N/A')}

Instructions:

Evaluate the Interpretation section using the following scoring criteria:

Part 1: Evaluation of the Interpretation of Risk Factors

Item 1.1 Integration – Clinical Relevance

0: No relevant risk factors mentioned. (Example: Omitting key factors such as intubation status or vital signs)

1: Bare mention of risk factors without context or proper interpretation. (Example: Simply listing "intubation" without linking it to the patient's risk outcome)

2: Basic identification of risk factors without explicit reference to SHAP values, or includes misinterpretation (e.g., normal variables are incorrectly identified as increasing risk). (Example: Listing intubation and SpO₂ as risk factors without showing their SHAP values or clarifying their true clinical impact)

3: Clear explanation that includes numerical SHAP values to identify risk factors. (Example: Explains that intubation increases risk by citing its SHAP value, but with limited integration of broader clinical context)

4: Detailed integration of numerical SHAP values with thorough clinical correlation and context, accurately relating both risk-enhancing and protective factors. (Example: Thoroughly explains how the high SHAP value for intubation and the low or negative SHAP values for normal variables combine to define the patient's overall risk profile)

Item 1.2 Integration – Clarity of Information

0: The interpretation is disorganized and unclear, lacking a coherent structure or logical flow.

1: The interpretation is partially clear with noticeable ambiguities that impede full understanding of the risk factors.

2: The interpretation is generally clear but only provides a basic structure without exploring potential causes of the risk factors.

3: The interpretation is clear and organized, with a logical flow and mention of at least one potential cause of the risk factors.

4: The interpretation is exceptionally clear and well-organized, seamlessly presenting multiple distinct potential causes of the risk factors.

Item 1.3 Mastery – Balance and Bias Assessment

0: One-sided – Only risk-enhancing factors are discussed, with no mention of protective factors.

1: Minimal balance – Protective factors are mentioned but are mischaracterized or insufficiently addressed (e.g., normal variables are erroneously noted as contributing to increased risk).

2: Moderately balanced – Both risk-enhancing and protective factors are mentioned, but the protective factors receive limited or imprecise emphasis (e.g., protective factors are noted but with slight misinterpretation).

3: Balanced – Both risk-enhancing and protective factors are clearly discussed, with a mild emphasis on protective factors and overall fair balance.

4: Well-balanced – A comprehensive and thorough discussion of both risk-enhancing and protective factors is provided, with detailed and accurate emphasis on the protective factors.

Item 1.4 Mastery – Uncertainty Acknowledgment

0: No acknowledgment of uncertainty, limitations, or assumptions is provided when they are clearly relevant (e.g., in moderate-to-high risk scenarios, failing to mention any potential variability or interaction among risk factors).

1: Minimal acknowledgment of uncertainty is present but lacks detail and does not explore how risk factors might interact (e.g., a brief, cursory note on possible influencing factors without further explanation).

2: A basic acknowledgment of uncertainty is made with a brief mention of limitations or assumptions, or in cases where the predicted risk is extremely low (<2% ICU mortality) the absence of detailed uncertainty discussion is acceptable (e.g., a low-risk scenario where the response neutrally states the risk without additional qualifiers).

3: A clear acknowledgment of uncertainty is provided, detailing potential interactions among risk factors and limitations in the interpretation (e.g., a response that explicitly discusses assumptions and outlines areas where further assessment might be needed).

4: A comprehensive acknowledgment of uncertainties is offered, with an in-depth discussion of assumptions, limitations, and potential for further deterioration (e.g., a detailed exploration of risk factors, their interactions, and the boundaries of the prediction model).

Item 1.5 Precision – Accuracy of Content

0: The interpretation is incorrect, with major misinterpretations of key risk factors and their impact.

1: The interpretation is mostly inaccurate, containing notable errors or misrepresentations of the clinical significance of the risk factors.

2: The interpretation is partially accurate, correctly identifying some risk factors but overemphasizing minor details or missing the overall clinical context.

3: The interpretation is mostly accurate, adequately reflecting the clinical severity of the risk factors with proper integration of the SHAP values.

4: The interpretation is fully accurate, thoroughly incorporating numerical SHAP values to clearly and precisely reflect the clinical severity and implications of each risk factor.

Item 1.6 Precision – Knowledge Depth and Currency

0: Superficial – The response lacks depth and fails to integrate current clinical evidence or detailed reasoning.

1: Limited – The response offers a basic, student-level interpretation with minimal integration of current clinical insights.

2: Adequate – The response demonstrates acceptable depth and understanding typical of a junior resident, though some nuances may be missing.

3: Good – The response shows in-depth reasoning with current clinical insight appropriate for a senior resident, clearly explaining key risk factors and their significance.

4: Expert-level – The response exhibits advanced, nuanced clinical understanding with comprehensive integration of the latest evidence, characteristic of an attending physician.

Item 1.7 Applicability – Patient-specific considerations

0: Superficial – The response ignores key patient-specific factors and provides a generic, non-tailored analysis.

1: Limited – The response mentions patient-specific details in a superficial manner without adequate explanation of their significance.

2: Adequate – The response identifies most risk factors but lacks depth in explaining how these factors interact specifically in the patient's context.

3: Good – The response clearly explains the major risk factors and their clinical impact with appropriate patient-specific considerations, though without extensive nuance.

4: Expert-level – The response provides a comprehensive and nuanced analysis that thoroughly integrates each patient-specific factor and their interactions, offering detailed clinical rationale

Item 1.8 Comprehensiveness – Complete Scope

0: Major omissions – The response omits many key risk factors, providing an incomplete picture.

1: Several missing – Multiple important risk factors are not addressed, resulting in a noticeably incomplete analysis.

2: Mostly complete – Most of the major risk factors are mentioned, but some relevant details or interactions are missing.

3: Comprehensive – Nearly all critical risk factors and their interactions are addressed, though with minor omissions in detail.

4: Exhaustive – Every relevant risk factor is clearly detailed, including nuanced interplay and critical conditions.

Item 1.9 Timeliness – Response Urgency

0: No appropriate urgency – The response fails to match the risk profile, neither identifying critical/life-threatening factors in high-risk cases nor reassuring stability in low-risk scenarios.

1: Minimal urgency – The response mentions risk factors but underemphasizes life-threatening conditions in high-risk cases or overstates them in stable situations.

2: Moderate urgency – The response recognizes risk factors, identifying potential life-threatening conditions in high-risk cases or confirming stability in low-risk scenarios without extraneous commentary.

3: High urgency – The response distinguishes key risk factors by prioritizing recognition of critical/life-threatening conditions in high-risk cases or confidently confirming stability in low-risk situations.

4: Critical calibration – The response flawlessly aligns with the risk profile by precisely identifying critical/life-threatening conditions in high-risk cases or accurately assuring stability in low-risk scenarios.

Provide a JSON output with the key "Interpretation" containing:

- "subtotal": <subtotal score for Interpretation>,

- "items": [{{ "item": "<item name>", "score": <score>, "comment": "<explanation>" }}, ...]

Ensure the JSON is valid.

**Part 2 System Prompt**

"You are an expert clinical evaluator with extensive clinical and evidence-based practice experience.\n"

Your task is to thoroughly analyze the LLM responses provided for the Interpretation and Examination sections and provide a detailed numerical scoring breakdown for the Examination evaluation."

**Part 2 User Prompt**

Patient Information:

{row.get('Patient', 'N/A')}

LLM Response for Interpretation:

{row.get('Interpretation', 'N/A')}

LLM Response for Examination:

{row.get('Examination', 'N/A')}

Instructions:

Evaluate the Examination section using the following scoring criteria:

Part 2: Evaluation of the Recommended Examinations

Item 2.1 Integration – Clear aim

0: No clear diagnostic aim (e.g., suggests tests that do not address the primary risk factors for ICU mortality)

1: Vague or minimal diagnostic aim with limited focus (e.g., recommends tests without explicitly linking them to the confirmation of underlying risk factors)

2: Moderately integrated diagnostic aims (e.g., recommends tests that address major risk factors but lack explicit prioritization or complete integration of diagnostic objectives)

3: Clear diagnostic aims with substantial integration (e.g., recommends tests clearly aimed at confirming risk factors with good linkage, though some aspects may not be fully comprehensive)

4: Fully integrated, explicit diagnostic aims (e.g., precisely prioritizes bedside, urgent, and advanced tests that explicitly target the confirmation and differentiation of ICU risk factors)

Item 2.2 Mastery – Correct clinical reasoning

0: Entirely misdirected clinical reasoning with recommendations that do not address the patient's major risk factors

1: Basic clinical reasoning with significant omissions, leading to incomplete or misaligned test recommendations

2: Partial clinical reasoning that covers some key aspects but contains notable gaps or inconsistencies

3: Solid clinical reasoning with appropriate test recommendations that address major risk factors, albeit with minor nuances missed

4: Exemplary clinical reasoning fully aligned with the patient's risk factors, providing a balanced, integrated, and comprehensive set of examinations

Item 2.3 Precision – Accuracy of Content

0: Incorrect recommendations with tests that are irrelevant or misleading for the patient's condition

1: Several inaccuracies in the test recommendations, with significant errors in clinical appropriateness

2: Partially accurate content with some tests appropriate while others are not fully aligned with clinical needs

3: Mostly accurate content with recommended tests that are clinically appropriate, though with minor issues or less optimal precision

4: Completely accurate content with all examinations precisely matching clinical needs and aligned with the patient's risk factors

Item 2.4 Applicability – Patient-specific Considerations

0: No relevance to patient specifics; recommendations are entirely generic and ignore individual patient factors

1: Minimal tailoring with superficial consideration of patient-specific factors, resulting in largely generic recommendations

2: Moderately tailored recommendations that address some key patient-specific risks but lack full personalization

3: Well-aligned with patient-specific risks, demonstrating thoughtful consideration of the patient's unique clinical profile

4: Fully personalized and contextually comprehensive recommendations that integrate all relevant patient-specific data in a nuanced manner

Item 2.5 Comprehensiveness – Complete Scope

0: Fails to include most evaluation components; only a single or minimal test is recommended, leading to an inadequate assessment

1: Poor coverage with multiple critical evaluations missing, reflecting a significantly incomplete workup

2: Moderate scope with partial evaluation coverage; includes several tests but lacks comprehensive detail or depth

3: Comprehensive scope with minor omissions; covers all major evaluations but may miss some nuanced details

4: Exemplary complete coverage of all evaluations, fully integrating bedside assessment, urgent labs, continuous monitoring, advanced imaging, and a clear conclusion aligned with the patient's risk profile

Item 2.6 Timeliness – Priority

0: No recognition of urgency or misalignment in the recommended test sequence, leading to inappropriate prioritization

1: Minimal attention to urgency with vague or poorly sequenced recommendations that do not match the patient's critical needs

2: Moderately reflects response urgency with some timely recommendations but lacking a clear, structured sequence

3: Accurately matches urgency needs with a logical sequence of immediate, urgent, and follow-up assessments, with only minor adjustments needed

4: Optimal alignment with the required time sequence, precisely prioritizing bedside assessment, urgent investigations, and additional monitoring in strict accordance with the patient's clinical status

Provide a JSON output with the key "Examination" containing:

- "subtotal": <subtotal score for Examination>,

- "items": [{{ "item": "<item name>", "score": <score>, "comment": "<explanation>" }}, ...]

Ensure the JSON is valid.

**Part 3 System Prompt**

"You are an expert clinical evaluator with extensive clinical and evidence-based practice experience.\n"

"Your task is to thoroughly analyze the LLM responses provided for the Interpretation, Examination, and Management sections and provide a detailed numerical scoring breakdown for the Management evaluation."

**Part 3 User Prompt**

Patient Information:

{row.get('Patient', 'N/A')}

LLM Response for Interpretation:

{row.get('Interpretation', 'N/A')}

LLM Response for Examination:

{row.get('Examination', 'N/A')}

LLM Response for Management:

{row.get('Management', 'N/A')}

Instructions:

Evaluate the Management section using the following scoring criteria:

Part 3: Evaluation of the Recommended Management

Item 3.1 Integration – Clear aims

0: The management plan fails to state any clear treatment aims; interventions are listed without reference to major risk factors or any organization.

1: The plan mentions basic treatment steps (e.g., resuscitation or acute care) in isolation without linking them to patient-specific risk factors, resulting in no unified strategy.

2: The response groups interventions (e.g., separating immediate resuscitation from acute care) but overall integration is weak, with only partial linkage between phases and a limited summary.

3: The plan outlines clear and mostly targeted aims by dividing management into distinct phases (resuscitation, acute management, and supportive measures) and addresses major risk factors, though the integration may be brief or only partially cohesive.

4: The response provides a fully integrated, cohesive management plan that explicitly links immediate resuscitation, acute intervention, and ICU supportive care into a unified strategy, with a concise and clear conclusion tying all aspects together seamlessly.

Item 3.2 Mastery – Correct clinical reasoning

0: The plan shows no valid clinical reasoning; it recommends generic measures without addressing the patient's urgent resuscitation or ICU needs.

1: The plan demonstrates limited, inconsistent clinical reasoning, addressing some resuscitation elements while omitting crucial acute and supportive management steps.

2: The plan exhibits moderate clinical reasoning by incorporating key elements like immediate resuscitation and acute measures but has notable gaps in comprehensive ICU supportive care.

3: The plan displays strong clinical reasoning with nearly complete integration of immediate, acute, and supportive care components, though with minor omissions.

4: The plan demonstrates exemplary, fully integrated clinical reasoning by thoroughly addressing resuscitation, acute intervention, ICU support, and providing a clear, succinct conclusion.

Item 3.3 Precision – Accuracy of Content

0: Recommendations are clinically inaccurate or irrelevant, failing to address the patient's primary risk factors.

1: Recommendations contain several inaccuracies, such as suggesting inappropriate interventions while neglecting key immediate treatments.

2: Recommendations are partially accurate, addressing some essential interventions but omitting significant details in supportive care.

3: Recommendations are mostly accurate, providing precise guidance for immediate and targeted interventions with only minor omissions.

4: Recommendations are completely accurate, offering detailed and precise interventions that comprehensively address immediate, acute, and supportive care tailored to the patient's condition.

Item 3.4 Applicability – Patient-specific Considerations

0: No tailoring is evident; the recommendations follow a standard protocol without considering the patient's unique clinical profile.

1: The recommendations are generic, offering standard advice with minimal or no adjustments based on patient-specific factors.

2: Some adjustments are made based on patient data, but the recommendations lack depth in customization to the patient's unique profile.

3: The recommendations are well-tailored, incorporating key patient-specific factors such as age, comorbidities, and current clinical status to inform management decisions.

4: The recommendations are highly customized, integrating detailed patient-specific information to precisely modify interventions and optimize outcomes.

Item 3.5 Comprehensiveness – Complete Scope

0: The management plan omits several key interventions, failing to address immediate resuscitation, acute management, ICU supportive care, or a unifying conclusion.

1: The management plan includes several recommendations, but multiple critical interventions are missing, resulting in an incomplete scope.

2: The management plan is mostly complete, covering most key interventions, yet it lacks sufficient detail or integration in some areas.

3: The management plan is comprehensive, addressing immediate resuscitation, acute management, and ICU supportive care with a coherent structure, though minor details may be underdeveloped.

4: The management plan is exhaustive, including every essential intervention with thorough detail and seamless integration across immediate, acute, and supportive phases of care.

Item 3.6 Timeliness – Priority

0: The management plan fails to address timeliness, lacking any indication of urgency or time-sensitive intervention sequencing.

1: The plan mentions urgency but provides minimal focus on critical risk factors and lacks a clearly structured time sequence.

2: The plan moderately prioritizes major risk factors with some indication of time-sensitive steps, but the overall sequence and urgency remain too simplistic.

3: The plan mostly prioritizes critical interventions with a clear time-sensitive ordering, though the sequence may not be fully detailed.

4: The plan exemplarily prioritizes critical management steps with an explicit, well-structured time sequence that clearly distinguishes immediate from subsequent interventions

"""

Provide a JSON output with the key "Management" containing:

- "subtotal": <subtotal score for Management>,

- "items": [{{ "item": "<item name>", "score": <score>, "comment": "<explanation>" }}, ...]

Ensure the JSON is valid.

**Part 4 & 5 System Prompt**

"You are an expert clinical evaluator with extensive clinical and evidence-based practice experience.\n"

"Your task is to thoroughly analyze the LLM responses provided for the Interpretation, Examination, Management, Follow_up, and Summary sections and provide a detailed numerical scoring breakdown for the Follow_up and Summary evaluations."

**Part 4 & 5 User Prompt**

Patient Information:

{row.get('Patient', 'N/A')}

LLM Response for Interpretation:

{row.get('Interpretation', 'N/A')}

LLM Response for Examination:

{row.get('Examination', 'N/A')}

LLM Response for Management:

{row.get('Management', 'N/A')}

LLM Response for Follow_up:

{row.get('Follow_up', 'N/A')}

LLM Response for Summary:

{row.get('Summary', 'N/A')}

Instructions:

Evaluate the Follow_up and Summary sections using the following scoring criteria:

Part 4: Evaluation of the Follow-up Plan

Item 4.1 Precision – Accuracy of Content

0: The follow-up plan is grossly inaccurate, missing essential monitoring elements, and lacks any actionable or time-sequenced structure.

1: The plan includes some monitoring elements but has major inaccuracies or omissions. It lacks a coherent time sequence and fails to address the patient's dynamic needs.

2: The plan is partially accurate and contains basic monitoring steps; however, it is overly simplistic and does not provide a clear, sequential timeline for continuous monitoring, acute reassessment, and stability follow-up.

3: The plan is accurate with mostly complete details. It outlines monitoring and reassessment with a reasonable time sequence across continuous, acute, and stability phases, though minor refinements in the sequential structure may be needed.

4: The plan is highly precise and comprehensive. It delivers detailed, sequential follow-up that includes continuous monitoring, clearly defined intervals for acute reassessment, and structured stability follow-up, fully addressing the patient's evolving clinical profile.

Item 4.2 Applicability – Patient-specific Considerations

0: The follow-up plan is completely generic with no incorporation of patient-specific details. It does not address unique factors such as advanced age, intubation, hypothermia, or other individualized risks.

1: The plan shows minimal patient-specific tailoring. It may mention standard monitoring elements but lacks clear reference to the patient's unique risk factors and does not integrate these with a sequential or time-based structure.

2: The plan includes basic patient-specific details—addressing some aspects of the patient's risk profile—but remains too simplistic. It fails to integrate a detailed, time-sequenced structure that fully tailors monitoring and interventions to the patient's dynamic needs.

3: The plan provides substantial customization to the patient's clinical profile. It includes tailored monitoring and reassessment intervals that acknowledge key risk factors (e.g., intubation, hypothermia, advanced age) with a reasonably clear time sequence, though some minor details may be omitted.

4: The plan is highly tailored and fully patient-specific. It delivers a comprehensive, sequential follow-up that incorporates all relevant patient factors and dynamically adjusts monitoring and interventions based on the patient's evolving clinical status.

Item 4.3 Timeliness – Timely Reassessment Intervals

0: The management plan fails to address timeliness, lacking any indication of urgency or time-sensitive intervention sequencing.

1: Very limited, vague priority details (e.g., Mentions monitoring but lacks clear timing or explicit reassessment and stability protocols)

2: Partial coverage of priority tiers (e.g., Specifies continuous monitoring and acute reassessment but omits structured stability follow-up details)

3: Mostly comprehensive, priority-based follow-up (e.g., Outlines continuous monitoring, timely acute reassessment, and basic stability follow-up with minor omissions)

4: Fully tailored, comprehensive priority follow-up (e.g., Precisely details hourly checks, acute reassessment intervals, and structured stability follow-up, perfectly matching patient need)

Part 5: Evaluation of the Summary

Item 5.1 Comprehensiveness – Summary and Overall Quality

0: The summary lacks structure, omits major risk factors, and provides no priority actions or concluding recommendations.

1: The summary includes minimal risk factors with vague details, mentions fewer than two priority actions (often unrelated to key risks), and has minimal structure without clear introduction or conclusion.

2: The summary partially integrates key risk factors with some patient risks, lists one to two priority actions, but lacks clear organization in introduction or conclusion with some important details missing.

3: The summary presents a mostly comprehensive, clearly structured overview with an introduction outlining major risk factors, includes three priority actions (though not perfectly enumerated), and provides a brief concluding statement.

4: The summary delivers an exemplary, highly structured synthesis that succinctly restates all key risk factors, provides a detailed introduction, explicitly lists three well-defined priority steps, and offers an insightful conclusion with all elements effectively integrated.

Provide a JSON output with the keys:

"Follow_up": {{

"subtotal": <subtotal score for Follow_up>,

"items": [{{ "item": "<item name>", "score": <score>, "comment": "<explanation>" }}, ...]

}},

"Summary": {{

"subtotal": <subtotal score for Summary>,

"items": [{{ "item": "<item name>", "score": <score>, "comment": "<explanation>" }}]

}}

Ensure the JSON is valid.

# Supplementary Tables

**Supplementary Table 1. Inter-rater agreement among three raters across report sections.**

| **Section** | **Human vs o3-mini** | **Human vs Claude** | **o3-mini vs Claude** |
| --- | --- | --- | --- |
| **Interpretation** | 0.915 | 0.879 | 0.855 |
| **Examinations** | 0.879 | 0.921 | 0.918 |
| **Management** | 0.977 | 0.928 | 0.906 |
| **Follow-up and Summary** | 0.937 | 0.914 | 0.907 |
| **Total** | **0.975** | **0.979** | **0.971** |

**Supplementary Table 2. Baseline characteristics of the study population**

| **Variable** | **Overall (N = 120)** |
| --- | --- |
| ***Demographics*** |  |
| Female | 51 (42.5%) |
| Age, years | 67.7 (16.6) |
| Weight, kg | 80.3 (26.7) |
| ***Admission type*** |  |
| Emergency admission | 82 (68.3%) |
| ***Comorbidities*** |  |
| Charlson Comorbidity Index | 6.6 (2.9) |
| Myocardial infarction | 30 (25.0%) |
| Congestive heart failure | 44 (36.7%) |
| Peripheral vascular disease | 4 (3.3%) |
| Cerebrovascular disease | 22 (18.3%) |
| Dementia | 6 (5.0%) |
| Chronic pulmonary disease | 27 (22.5%) |
| Rheumatic disease | 5 (4.2%) |
| Peptic ulcer disease | 6 (5.0%) |
| Mild liver disease | 25 (20.8%) |
| Diabetes without complications | 32 (26.7%) |
| Diabetes with complications | 23 (19.2%) |
| Paraplegia | 12 (10.0%) |
| Renal disease | 32 (26.7%) |
| Malignant cancer | 14 (11.7%) |
| Severe liver disease | 18 (15.0%) |
| Metastatic solid tumor | 10 (8.3%) |
| ***Hospital course*** |  |
| Hospital LOS, days | 15.8 (18.4) |
| ICU LOS, days | 4.5 (5.9) |
| ***Vital signs at ICU admission*** |  |
| Temperature, °C | 36.7 (0.8) |
| Heart rate, bpm | 94.6 (22.6) |
| Respiratory rate, /min | 22.6 (6.6) |
| SpO₂, % | 96 (5) |
| Systolic BP, mmHg | 120 (30) |
| Diastolic BP, mmHg | 72 (21) |
| Mean BP, mmHg | 85 (23) |
| GCS | 15 (14-15) |
| ***Outcomes*** |  |
| Hospital mortality | 30 (25.0%) |
| ICU mortality | 21 (17.5%) |
| 30-day mortality | 37 (30.8%) |
| 90-day mortality | 42 (35.0%) |
| 180-day mortality | 47 (39.2%) |
| 1-year mortality | 51 (42.5%) |

Values are presented as mean (SD), median (IQR), or n (%). Percentages are calculated based on available (non-missing) data for each variable.

BP, blood pressure; GCS, Glasgow Coma Scale; ICU, intensive care unit; LOS, length of stay; SD, standard deviation; SpO₂, peripheral oxygen saturation.

**Supplementary Table 3. Baseline characteristics of the study population**

| **Variable** | **Overall (N = 360)** |
| --- | --- |
| ***Demographics*** |  |
| Female | 141 (39.2%) |
| Age, years | 67.1 (16.2) |
| Weight, kg | 80.6 (24.4) |
| ***Admission type*** |  |
| Emergency | 262 (72.8%) |
| ***Comorbidities*** |  |
| Charlson Comorbidity Index | 6.7 (3.1) |
| Myocardial infarction | 64 (17.8%) |
| Congestive heart failure | 119 (33.1%) |
| Peripheral vascular disease | 48 (13.3%) |
| Cerebrovascular disease | 90 (25%) |
| Dementia | 28 (7.8%) |
| Chronic pulmonary disease | 67 (18.6%) |
| Rheumatic disease | 13 (3.6%) |
| Peptic ulcer disease | 24 (6.7%) |
| Mild liver disease | 56 (15.6%) |
| Diabetes without complications | 75 (25.8%) |
| Diabetes with complications | 60 (16.7%) |
| Paraplegia | 36 (10%) |
| Renal disease | 98 (27.2%) |
| Malignant cancer | 72 (20%) |
| Severe liver disease | 36 (10%) |
| Metastatic solid tumor | 42 (11.7%) |
| ***Hospital course*** |  |
| Hospital LOS, days | 17.4 (19.6) |
| ICU LOS, days | 5.3 (6.9) |
| ***Vital signs at ICU admission*** |  |
| Temperature, °C | 36.7 (0.9) |
| Heart rate, bpm | 93.5 (23) |
| Respiratory rate, /min | 22.4 (6.5) |
| SpO₂, % | 96 (4) |
| Systolic BP, mmHg | 123 (26) |
| Diastolic BP, mmHg | 72 (19) |
| Mean BP, mmHg | 88 (22) |
| GCS | 15 (14-15) |
| ***Outcomes*** |  |
| ICU mortality | 49 (13.6%) |
| Hospital mortality | 89 (24.7%) |
| 30-day mortality | 107 (29.7%) |
| 90-day mortality | 133 (36.9%) |
| 180-day mortality | 139 (38.6%) |
| 1-year mortality | 153 (42.5%) |

Values are presented as mean (SD), median (IQR), or n (%). Percentages are calculated based on available (non-missing) data for each variable.

BP, blood pressure; GCS, Glasgow Coma Scale; ICU, intensive care unit; LOS, length of stay; SD, standard deviation; SpO₂, peripheral oxygen saturation.

# Supplementary Figures

**Supplementary Figure 1. Study workflow diagram showing dataset allocation for machine learning model development and large language model evaluation.**


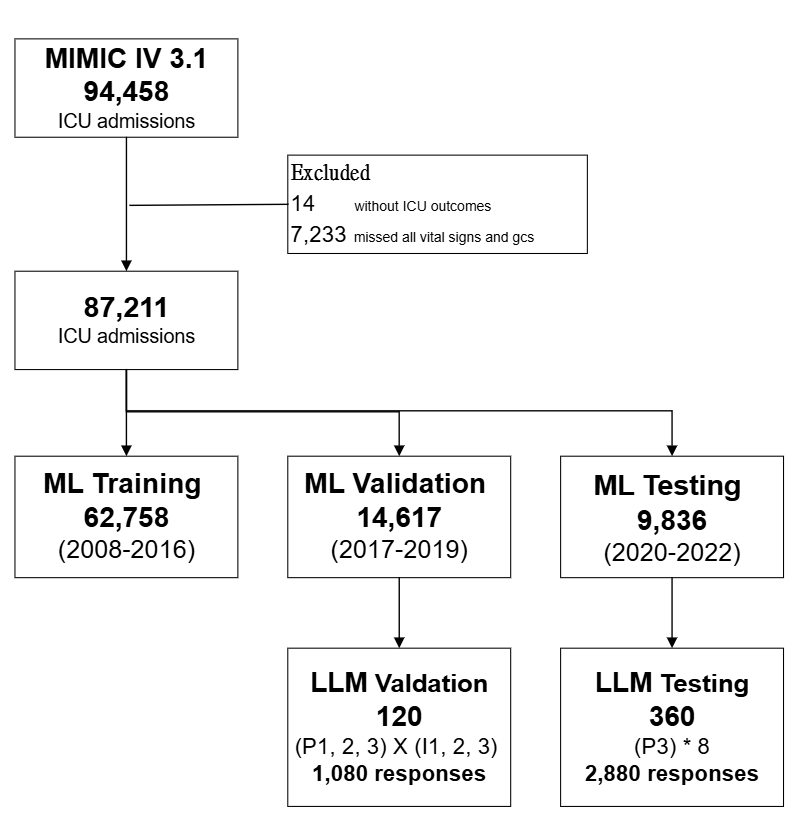


A total of 94,458 ICU admissions from MIMIC-IV 3.1 were screened, and 87,211 were included after excluding 14 admissions without ICU outcomes and 7,233 admissions with missing vital signs and Glasgow Coma Scale data. The included admissions were split by time into three datasets: ML training (n = 62,758; 2008 to 2016), ML validation (n = 14,617; 2017 to 2019), and ML testing (n = 9,836; 2020 to 2022). From the ML validation set, 120 patients were sampled for prompt optimization. From the ML testing set, 360 patients were sampled for the final LLM evaluation. GCS, Glasgow Coma Scale; ICU, intensive care unit; LLM, large language model; ML, machine learning; MIMIC, Medical Information Mart for Intensive Care.

**Supplementary Figure 2. Correlation between Claude 3.7 Sonnet scores and Human or o3-mini scores across evaluation sections**


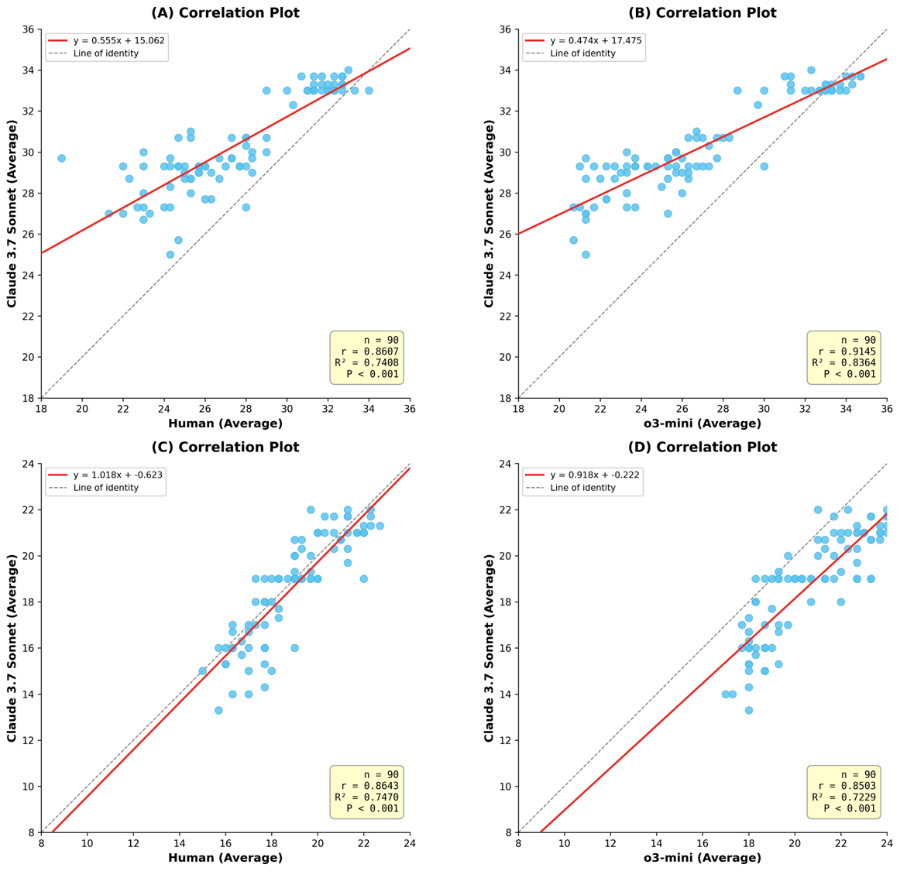


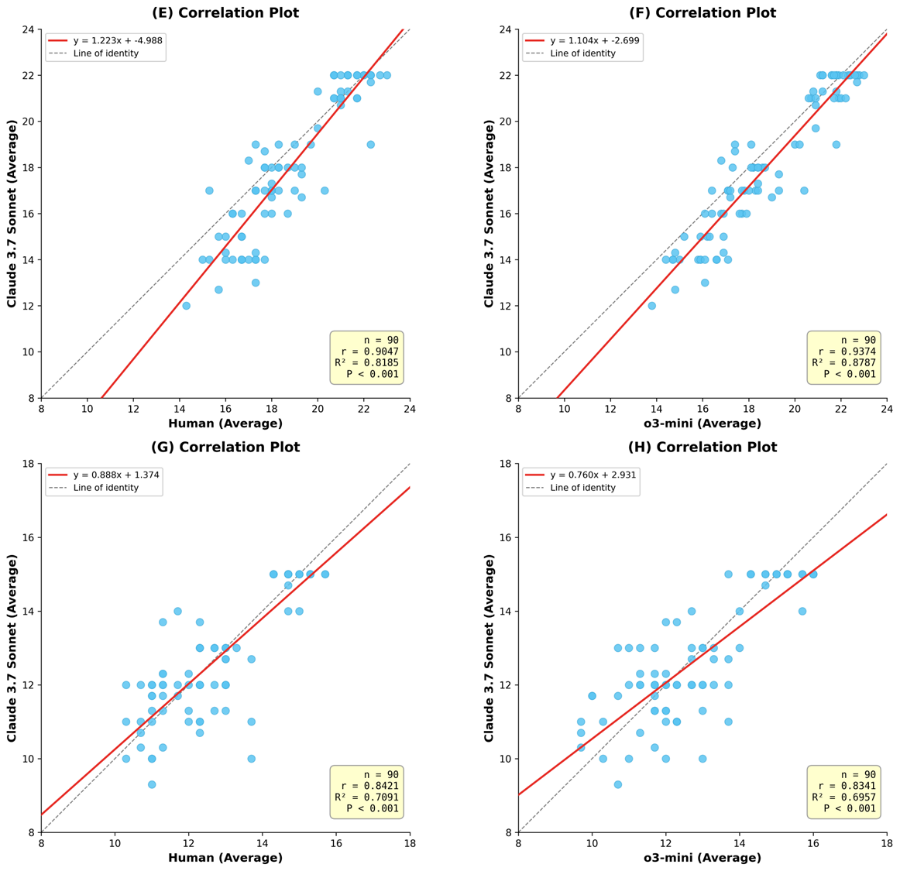


Scatter plots show the correlation between average scores assigned by Claude 3.7 Sonnet (y-axis) and those assigned by Human evaluators or o3-mini (x-axis) for 90 AI-generated ICU clinical decision support reports. The red solid line represents the linear regression line with the equation shown in the legend. The gray dashed line represents the line of identity (y = x). The yellow box in each panel displays the sample size (n), Pearson correlation coefficient (r), coefficient of determination (R²), and P value. Panels (A), (C), (E), and (G) compare Claude 3.7 Sonnet with Human scores, while panels (B), (D), (F), and (H) compare Claude 3.7 Sonnet with o3-mini scores. (A) and (B): Interpretation of risk factors. (C) and (D): Examinations. (E) and (F): Management. (G) and (H): Follow-up and summary. Data points above the line of identity indicate higher scores by Claude 3.7 Sonnet relative to the comparator; data points below indicate lower scores.
